# Gene knockdown via electroporation of short hairpin RNAs in embryos of the marine hydroid *Hydractinia symbiolongicarpus*

**DOI:** 10.1038/s41598-020-69489-8

**Published:** 2020-07-30

**Authors:** Gonzalo Quiroga-Artigas, Alexandrea Duscher, Katelyn Lundquist, Justin Waletich, Christine E. Schnitzler

**Affiliations:** 1 Whitney Laboratory for Marine Bioscience, University of Florida, St. Augustine, FL 32080 USA; 2 Department of Biology, University of Florida, Gainesville, FL USA

**Keywords:** Biological techniques, Developmental biology

## Abstract

Analyzing gene function in a broad range of research organisms is crucial for understanding the biological functions of genes and their evolution. Recent studies have shown that short hairpin RNAs (shRNAs) can induce gene-specific knockdowns in two cnidarian species. We have developed a detailed, straightforward, and scalable method to deliver shRNAs into fertilized eggs of the hydrozoan cnidarian *Hydractinia symbiolongicarpus* via electroporation, yielding effective gene-targeted knockdowns that can last throughout embryogenesis. Our electroporation protocol allows for the transfection of shRNAs into hundreds of fertilized *H. symbiolongicarpus* eggs simultaneously with minimal embryo death and no long-term harmful consequences on the developing animals. We show RT-qPCR and detailed phenotypic evidence of our method successfully inducing effective knockdowns of an exogenous gene (*eGFP*) and an endogenous gene (*Nanos2*), as well as knockdown confirmation by RT-qPCR of two other endogenous genes. We also provide visual confirmation of successful shRNA transfection inside embryos through electroporation. Our detailed protocol for electroporation of shRNAs in *H. symbiolongicarpus* embryos constitutes an important experimental resource for the hydrozoan community while also serving as a successful model for the development of similar methods for interrogating gene function in other marine invertebrates.

## Introduction

**Figure 1:**
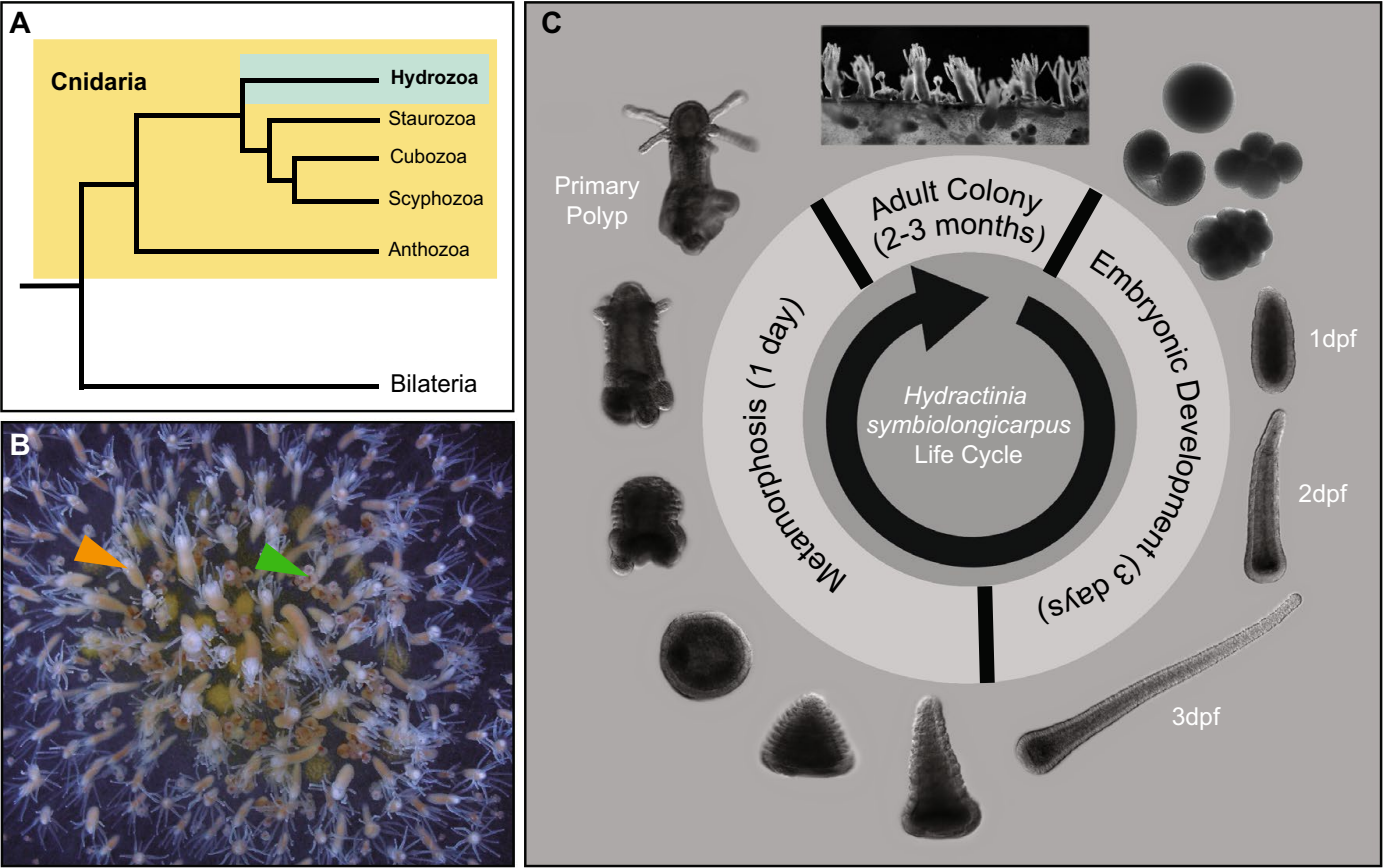
The hydrozoan cnidarian *Hydractinia symbiolongicarpus*. (**A**) Cladogram depicting the phylogenetic position of Cnidaria as sister group of Bilateria, as well as the phylogenic relationships between Hydrozoa and other cnidarian groups, based on Kayal et al.^50^. (**B**) Photo of a female adult colony of *H. symbiolongicarpus*. Orange arrowhead points to a feeding polyp and green arrowhead points to a reproductive polyp. (**C**) *H. symbiolongicarpus* life cycle. Images show key stages of embryonic development as well as stages of metamorphosis from a larva to a primary polyp and ultimately the adult colony. Mature female and male colonies spawn hundreds to thousands of gametes daily following a light cue. Spawned eggs are fertilized in the water column, and proceed through embryonic development^6^. About 3 days post-fertilization (3 dpf), fully-developed larvae are competent to receive a natural or artificial stimulus that will induce settlement and metamorphosis^51,52^. Metamorphosis is completed within 24 h, and this process transforms a mouthless larva into a feeding primary polyp. The animal then expands clonally by stolonal elongation and asexual budding of new polyps. The polyps are interconnected by a stolonal mat which holds gastrovascular canals, enabling food distribution and transfer of stem cells throughout the growing colony^53^. Two to three months after metamorphosis, reproductive polyps start to bud, allowing the colony to reproduce sexually.

Hydrozoans are members of the phylum Cnidaria, a group that holds a phylogenetic position as sister to all bilaterian animals^1^ (Fig. 1A), and thus can provide insights into the origins of key bilaterian features. Research involving hydrozoans has greatly contributed to our understanding of crucial cellular and developmental processes, as well as their evolution^2^. Within the Hydrozoa, members of the genus *Hydractinia* have been used as experimental research organisms for more than a century^3–5^. *Hydractinia* is a dioecious, marine, colonial hydroid that is well-suited for lab culturing and rearing. Its small size and transparency make it tractable and convenient for manipulation and microscopic imaging. Moreover, its embryonic development has been well-described^6^, allowing for targeted developmental studies. A fundamental characteristic of *Hydractinia* is that it maintains a population of stem cells called interstitial stem cells (or ‘i-cells’) that provides progenitors to both somatic and germ cell lineages in a continuous manner throughout its lifetime, allowing for remarkable regenerative capabilities and longevity^3,4,7^. The availability of a sequenced genome, and the range of functional genomic tools currently available make *Hydractinia* a rapidly maturing cnidarian system that is allowing researchers to explore a wide array of biological topics, ranging from stem cells and regeneration to developmental biology and self-recognition (allorecognition)^3–5,8^. In this study, we focused on a new method to silence genes in the species *H. symbiolongicarpus* (Fig. 1B), whose life cycle is shown in Fig. 1C. The relatively short and accessible life cycle of *H. symbiolongicarpus* allows for experimental manipulation of all life stages.

In the last decade, a variety of techniques to study gene function have become available for *Hydractinia*. Most recently, genetic engineering (both gene knockouts^9^ and knockins^10^) has been achieved in *Hydractinia* by means of CRISPR/Cas9 embryo microinjection. Prior to this, the use of antisense RNA-mediated gene silencing to induce gene-specific knockdown had been frequently employed in *Hydractinia*, via morpholino microinjection of embryos^11^ and double-stranded RNA (dsRNA) soaking of embryos and polyps^7,12–15^.

dsRNAs exploit the endogenous RNA interference (RNAi) machinery of the cells and have been used as molecular tools to transiently lower (or knock down) the transcript levels of a particular target gene for decades^16^. In contrast, short hairpin RNAs (shRNAs) are small, synthetic dsRNA molecules connected by a hairpin loop that can be used instead of longer dsRNAs to knock down target genes via RNAi^17^. shRNAs are processed similarly to precursor microRNAs (pre-miRNAs) through the endogenous RNAi pathway of transfected cells^18^. shRNAs have been widely used to induce gene knockdowns in mammalian cell culture^19^, in vivo in mammalian models^20^, and in model systems such as the fruit fly^21^ and zebrafish^22^. This shRNA-based knockdown approach has recently been used to target a small number of genes in two cnidarian species^23–25^, capitalizing on the specificity of the cnidarian miRNA pathway^23,26^ to achieve robust knockdowns. Two studies with the anthozoan cnidarian *Nematostella vectensis* demonstrated gene-specific knockdowns by delivering in vitro*-*synthesized shRNAs inside unfertilized eggs either via microinjection^23^ or electroporation^24^. In a more recent study using *H. symbiolongicarpus*, shRNAs microinjected in fertilized eggs successfully yielded targeted gene knockdown^25^, making shRNAs a promising tool to silence genes in *Hydractinia*.

One of the factors currently limiting the wider application of shRNAs as a gene silencing strategy in *Hydractinia* and other hydrozoan embryos is the lack of a method for fast, scalable, and efficient shRNA delivery. Microinjection as a delivery method is limited by the number of eggs that can be injected and the number of conditions that can be examined in the course of a single experiment. This is particularly problematic when working with fertilized eggs, which begin to cleave in under an hour after fertilization occurs^6^. Delivery via soaking requires constant incubation with relatively high concentrations of the antisense molecules, an approach that can be expensive and may not be consistently effective due to potential cell permeability changes throughout an experiment. As an alternative to these delivery methods, electroporation has been widely used by developmental biologists to transfect embryos from different phyla with a range of biomolecules^27^.

Electroporation is a physical transfection method that temporarily increases cell membrane permeability when submitted to electric field pulses^28^. The major advantage of this delivery method is that it is very fast, allowing for the transfection of many hundreds of eggs/cells at one time, as well as allowing researchers to examine a larger number of experimental conditions per assay. One of the challenges of electroporation is balancing efficient transfection and high survivorship, which involves finding conditions that allow the animals to survive the transfection process. The specific electroporation parameters (voltage, number of pulses, and pulse length), therefore, must be optimized for each species, life cycle stage, and biomolecule type. In addition to the electroporation of shRNAs in *N. vectensis* eggs^24^, this method has been applied to a few other cnidarians, including adult polyps of the freshwater hydrozoan *Hydra magnipapillata,* which have been successfully transfected with plasmids, dsRNAs, and small interfering RNAs (siRNAs)^29,30^, as well as polyps of the scyphozoan *Aurelia aurita* which were electroporated with dsRNAs^31^.

Here, we describe a detailed, optimized, and accessible method for simultaneously transfecting hundreds of one-cell stage embryos with shRNAs via electroporation in *H. symbiolongicarpus*—the first report of the successful application of this methodology in embryos from any hydrozoan cnidarian species. We demonstrate that this procedure can successfully induce the knockdown of both exogenous and endogenous targeted genes, and show for *eGFP* and *Nanos2* genes that their knockdown lasts throughout embryonic development and potentially further. We provide visual confirmation of the successful delivery of shRNAs inside embryonic cells upon electroporation, as well as a 28-day time series experiment showing the fluorescence dynamics over time of *eGFP* shRNA-electroporated animals and proof that there are no long-term harmful consequences on the shRNA-transfected animals. This new method will enable researchers interested in different facets of hydrozoan biology to study the function of targeted genes in a simple and scalable manner and represents a significant experimental advance that will be of benefit to the broader community of researchers working with a variety of cnidarian and other marine invertebrate species.

## Methods

### Animal care and breeding

*Hydractinia symbiolongicarpus* adult colonies were maintained at the University of Florida’s Whitney Laboratory for Marine Bioscience. Colonies were grown on glass microscope slides and cultured in 38-L tanks filled with artificial seawater (Instant Ocean-Reef Crystals) at 30 ppt and kept at 18–20 °C under a 10 h/14 h light/dark regime. Animals were fed three times a week with 3-day-old brine shrimp nauplii cultured at 25 °C (Premium Grade, Brine Shrimp Direct), enriched with Shellfish Diet (Reed Mariculture) 1 day after hatching, and twice a week with an oyster puree made from freshly caught, shucked, and blended oysters (stored at − 20 °C). *eGFP* experiments were performed with embryos from a cross of a *H. symbiolongicarpus Eef1alpha* > *eGFP* male transgenic line (354–3) and a female wildtype line (295–8). All other experiments were performed with embryos from a cross of a male wildtype line (291–10) with a female wildtype line (295–8).

Spawning of male and female gametes was induced by light stimulation. Sperm and eggs were mixed together to allow fertilization (detailed procedure in Supplementary Info S1). All embryos were allowed to develop into planula larvae in 100 mm × 15 mm glass petri dishes. When necessary, 72 hpf (hours post-fertilization) larvae were induced to metamorphose by incubating them in 116 mM CsCl solution in MFSW (Millipore-filtered seawater) for 3 h, as previously described^8^. They were then washed twice in MFSW, and finally transferred with gelatin-coated glass pipettes onto 75 × 25 mm glass microscope slides for settlement. Primary polyps that were kept for longer than 2 days post-metamorphosis were mouth-fed smashed brine shrimp every other day.

### shRNA design and synthesis

shRNA design was generally performed as previously described for the cnidarian *N. vectensis*^23,24^. A detailed protocol of our design strategy with examples can be found in Supplementary Info S2. Sequence data from the *H. symbiolongicarpus* male wildtype strain (291–10) is available via the *Hydractinia* Genome Project Portal, at https://research.nhgri.nih.gov/hydractinia/ to aid in the design. The DNA templates for in vitro transcription of shRNAs are composed of the T7 RNA polymerase promoter at the 5′ end, followed by the forward strand (siRNA) targeting the gene of interest, a hairpin loop linker with the sequence TTCAAGAGA, the reverse complement of the siRNA, and two additional thymidines at the 3′ end to mimic the endogenous pre-miRNA structure. Forward and reverse oligos of 66 bases in length corresponding to the DNA templates for in vitro transcription of shRNAs (Supplementary Table S1) were ordered from ThermoFisher Scientific, resuspended in nuclease-free H_2_O to a concentration of 100 μM and stored at − 20 °C.

shRNA synthesis was carried out as previously described^24^ with minor modifications. Briefly, dsDNA templates for in vitro transcription of shRNAs were constructed by mixing 2 μl of each of the two 66-base oligos (forward and reverse;100 μM each) with 16 μl of nuclease-free H_2_O, followed by a 2 min incubation at 98 °C and a 10 min incubation at RT (room temperature). In vitro transcription of shRNAs was performed using the AmpliScribe T7-Flash Transcription Kit (Lucigen). The transcription reaction was allowed to continue at 37 °C for 5–7 h. An incubation with DNase at 37 °C for 30 min was included at this point to eliminate the DNA template. Newly synthesized shRNAs were then purified using the Direct-zol RNA MiniPrep Kit (Zymo Research), eluted in 35 μl of nuclease-free H_2_O and quantified with a NanoDrop 2000 spectrophotometer (ThermoFisher Scientific). Normally, two synthesis reactions per shRNA were performed in parallel and pooled through the same purification column to attain higher concentrations of shRNAs. Purified shRNAs were stored at − 80 °C for up to 4 months.

### Electroporation procedure

**Figure 2:**
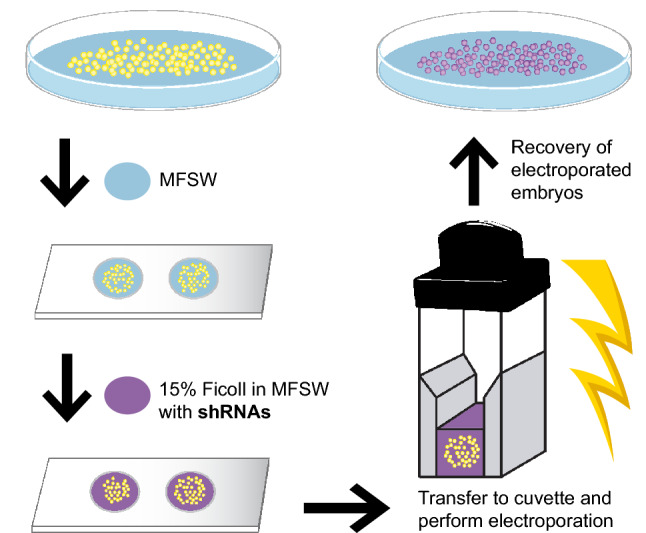
Overview of the shRNA electroporation procedure for *H. symbiolongicarpus* embryos. One-cell stage embryos are collected in a petri dish and transferred in a small volume of Millipore-filtered seawater (MFSW—blue) into wells of a depression slide. MFSW in each well is removed and replaced by 100 μl of the electroporation solution, consisting of 15% Ficoll-400 in MFSW containing the shRNAs (purple). The embryos in the electroporation solution are then transferred into an electroporation cuvette. The cuvette is placed inside the safety stand of the ECM 830 Square wave electroporation system (BTX) and electroporation of the embryos with the chosen parameters is carried out. Electroporated embryos are then carefully transferred to a petri dish with MFSW for their recovery and future phenotypic analyses. A detailed protocol can be found in Supplementary Info S1.

A detailed, step-by-step protocol can be found in Supplementary Info S1. Briefly, 300–900 fertilized eggs (also referred to as one-cell stage embryos) were transferred to a well in a glass depression slide and seawater was removed and replaced with 100 μl of the electroporation mixture (15% Ficoll-400 in MFSW containing shRNAs; Dextran; or nuclease-free H_2_O at given experimental concentrations). Ficoll-400 is a large synthetic polysaccharide that has been previously shown to make embryos float, allowing a more homogeneous voltage delivery, when mixed with seawater at 15% concentration^24^. Next, embryos were transferred into an electroporation cuvette (2 mm gap) and placed inside the safety stand connected to the ECM 830 square wave system (BTX). Embryos were then subjected to experimental electroporation parameters. When more than one pulse was delivered, the time span between pulses was always kept at 0.5 s. After a few seconds, electroporated embryos were gently transferred to a 100 mm × 15 mm glass petri dish filled with MFSW and left undisturbed to recover and develop for several hours (Fig. 2). Dead and non-developing embryos were removed once a day, including on the day of electroporation, to allow for better development of the survivors. MFSW was also changed daily. All embryos were kept at ~ 18 °C throughout experimental procedures and subsequent development. Non-electroporated (NE) controls were prepared several times by soaking the embryos in the *eGFP* shRNA mixture at 300 ng/μl each for the same length of time as the electroporation procedure (~ 3 min). In all cases, we observed that the NE control yielded no obvious fluorescence reduction or *eGFP* knockdown. Therefore, to avoid wasting shRNAs, we used the same proportions of nuclease-free H_2_O instead of shRNAs in the NE controls for all subsequent experiments with *eGFP* or the targeted endogenous genes.

### Light and fluorescence imaging

**Figure 3:**
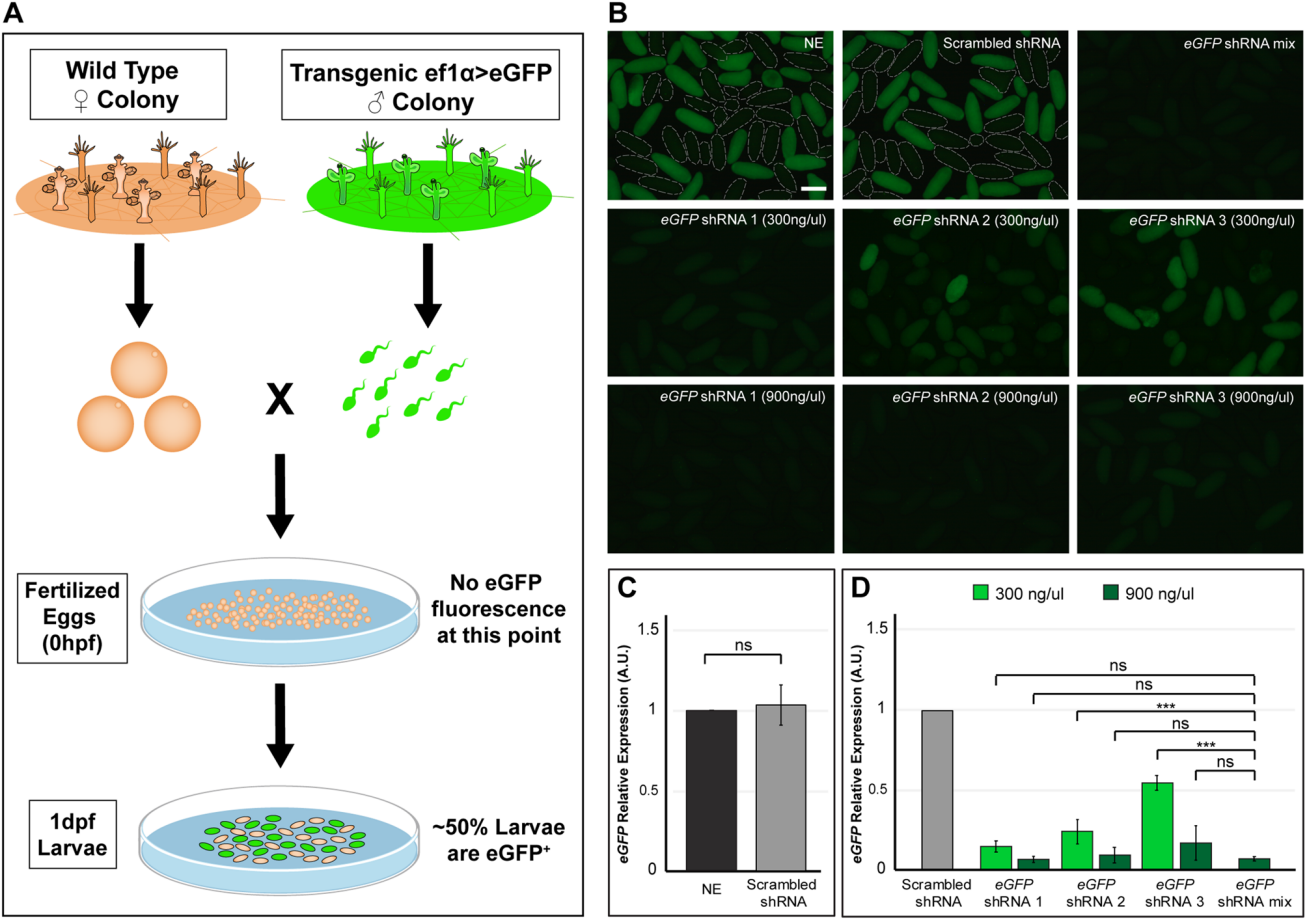
Visualization and quantification of *eGFP* knockdown. (**A**) Schematic depicting the cross performed leading to ~ 50% of eGFP^+^ larvae in the offspring due to Mendelian inheritance. This cross was carried out for all experiments involving the *eGFP* gene. (**B**) Representative fluorescence images of 1 dpf larvae for each of the nine experimental conditions shown. Note the qualitative reduction in eGFP fluorescence in the *eGFP* shRNA-electroporated conditions when compared to control larvae, with the *eGFP* shRNA mixture (mix) condition and the individual shRNAs at 900 ng/µl yielding the highest reductions. eGFP^−^ larvae are outlined with dashed lines in the NE and scrambled shRNA controls for better clarity. *NE* non-electroporated. Scale bar 200 μm. (**C**) RT-qPCR results showing equivalent expression levels of *eGFP* mRNA in control samples. Scrambled shRNA *eGFP* expression levels were quantified relative to the NE control. (**D**) RT-qPCR results showing a clear reduction in the *eGFP* mRNA expression levels in all *eGFP* shRNA-electroporated conditions when compared to the scrambled shRNA control. When comparing the *eGFP* shRNA mixture condition with any of the three *eGFP* shRNAs electroporated separately at a concentration of 300 ng/μl each, the mixture yielded the lowest *eGFP* transcript level of all, significantly lower than *eGFP* shRNA 2 and 3 (p value ≤ 0.01), although not significantly lower than *eGFP* shRNA 1*.* When comparing the *eGFP* shRNA mixture condition with any of the three *eGFP* shRNAs electroporated separately at a concentration of 900 ng/μl each, all conditions yielded similarly low levels of *eGFP* transcripts, indicating comparable knockdown efficiencies between these four conditions. For all *eGFP* shRNA experimental conditions, *eGFP* expression levels were quantified relative to the scrambled shRNA control. All RNA samples used for RT-qPCR analyses were extracted from 1 dpf larvae. Bar heights represent mean values of at least three independent experiments and error bars show standard errors of the mean. *Ns* non-significant; ***p value ≤ 0.01.

**Figure 4:**
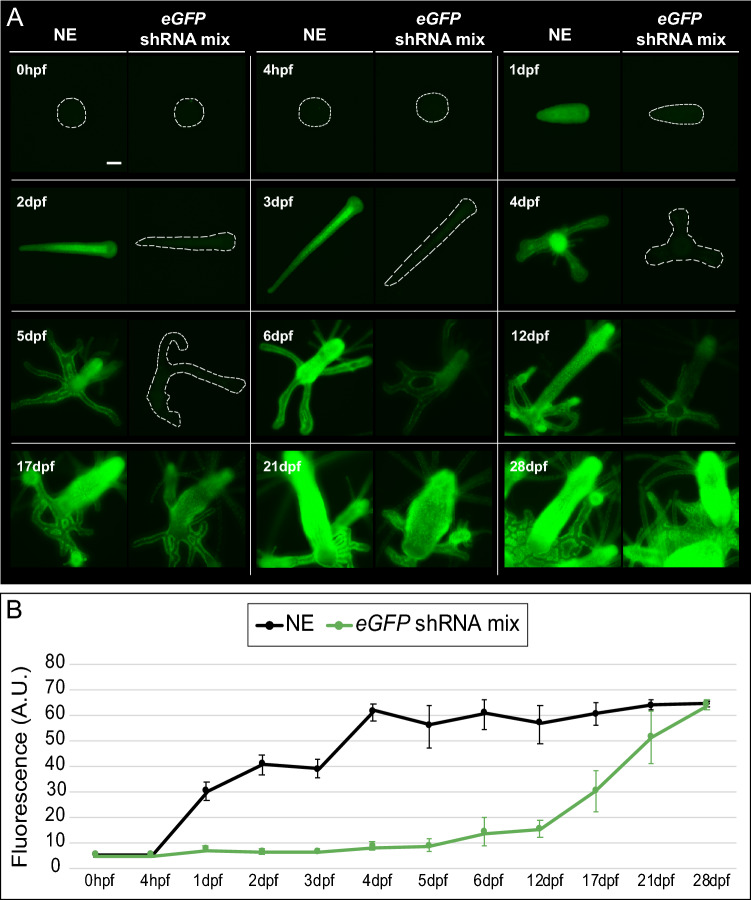
Fluorescence dynamics of *eGFP* knockdown over time. (**A**) Representative fluorescence images of embryonic and polyp stages are shown for each selected timepoint and condition. For clarity, embryos and polyps are outlined with dashed lines in all cases where eGFP fluorescence is not obvious. Scale bar 100 μm. (**B**) Fluorescence quantification over time. ~ 50% of NE control animals started appearing fluorescent by 1 dpf, and their fluorescence levels reach an upper threshold (fluorescence saturation) by 4 dpf. In contrast, the *eGFP* shRNA mixture-electroporated animals show very low fluorescence levels throughout embryonic development and during the first 2 days of primary polyp growth. At the 6 dpf timepoint, the fluorescence levels start to increase in ~ 50% of the polyps, and it took up to 28 dpf for the fluorescence levels to be equivalent to the NE control. Dots represent mean values and error bars show standard deviations (n = 10–16 for embryonic samples; n = 20–50 for polyp samples). *A.U*. arbitrary units.

Images taken for survivorship and eGFP (enhanced green fluorescent protein) fluorescence analyses as well as for Supplementary Figs. S1 and S2 were taken with a digital camera (Canon DS126201) attached to a stereo microscope (Zeiss, Discovery.V8). Light and fluorescence images shown in Figs. 1, 3, 4, 6 and Supplementary Fig. S3 were taken with a Rolera EM-C^2^ high-speed camera (QImaging) attached to a fluorescence microscope (Zeiss, Imager.M2). Identical scanning parameters (i.e., magnification and exposure time) were used for all conditions for each independent experiment.

### Counting Dextran^+^/eGFP^+^ larvae and survivorship analyses

All image processing was done using ImageJ software^32^. To count Dextran^+^ and eGFP^+^ larvae, the image background was enhanced identically for all images, and individuals were highlighted using identical thresholding for all conditions, separated using a watershed filter setting, and counted with the ‘Analyze Particles’ tool (Supplementary Figs. S1, S2). To assess survivorship, the same procedure was carried out, without background enhancement, for all life cycle stages assessed (Supplementary Table S2).

### eGFP fluorescence quantification

To quantify eGFP fluorescence, we used ImageJ^32^ to draw squares of 50 × 50 pixels in the mid-region of embryos and polyp body columns. Then, using the “Measure” option, we recovered the “RawIntDen” values of the eGFP channel, which refers to the sum of the pixel intensity values in the selected region of interest. These values divided by 1,000,000 correspond to the arbitrary units (A.U.) in Fig. 4B.

### RT-qPCR

To quantify shRNA knockdown of the targeted genes, total RNA was extracted with the RNAqueous-Micro Total RNA Isolation Kit (Ambion) at different timepoints from larvae or primary polyps, depending on the experiment. For each condition from every experiment, between 250 and 700 individuals were collected for RNA extraction. Samples were then treated with DNase according to manufacturer’s recommendations, and the RNA was reverse transcribed with random primers using the High Capacity cDNA Reverse Transcription kit (Applied Biosystems). qPCR analyses were performed using PowerUp SYBR Green Master Mix (Applied Biosystems) and a LightCycler 480 instrument (Roche). For each gene and condition, expression levels were derived from three amplification reactions, and normalized to housekeeping gene expression, either to *18S* rRNA (*eGFP* experiments) or to *Eef1alpha* (endogenous gene experiments). The delta-delta-ct method was used for quantification of transcript levels from experimental conditions relative to scrambled shRNA or NE (non-electroporated) controls. These relative expression levels are shown as arbitrary units (A.U.) in all figures. At least three independent biological replicates were performed for each experiment. Primer sequences can be found in Supplementary Table S1.

### Dig-shRNA synthesis and tyramide signal amplification

Digoxigenin-labeled shRNAs (Dig-shRNA) were synthesized as described above, using a nonspecific scrambled sequence as dsDNA template, and adding 0.5 μl UTP and 1.5 μl Digoxigenin-11-UTP (Roche) in the synthesis reaction.

**Figure 5:**
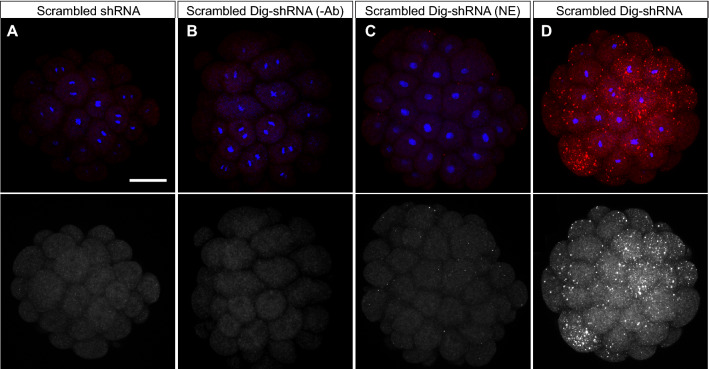
Visual confirmation of shRNA delivery into embryos via electroporation. Representative images of 4 hpf embryos are shown for each experimental condition. All images were projected from confocal z-stacks of ~ 20 μm. Hoechst staining of DNA is shown in blue (note some embryonic cells undergoing mitosis) and rhodamine-tyramide in red. The bottom row shows the single red channel in black and white for each condition to enhance contrast. In all cases, shRNAs were delivered at a concentration of 900 ng/μl. (**A**) Scrambled shRNA-electroporated embryo for which tyramide signal amplification (TSA) was carried out. (**B**) Digoxigenin-labeled scrambled shRNA-electroporated embryo for which TSA was performed but peroxidase-labeled anti-DIG antibody (Ab) was not added. Note the complete lack of rhodamine-tyramide signal in **A**,**B** controls. (**C**) Embryo that was soaked with digoxigenin-labeled scrambled shRNA for the length of an electroporation procedure (~ 3 min) but was not electroporated, and for which the TSA reaction was carried out. Note that some dots appear with the rhodamine-tyramide signal, mostly from shRNAs stuck on the surface but not inside the cells (see Supplementary Video S3). (**D**) Digoxigenin-labeled scrambled shRNA-electroporated embryo for which TSA was performed. Notice the intense fluorescence generated from the rhodamine-tyramide signal, largely coming from shRNAs that were delivered inside the one-cell stage embryonic stage via electroporation, and which stayed inside the cells throughout the first stages of cleavage (see Supplementary Video S4). Scale bar 50 μm.

Embryos were electroporated as described above with Dig-shRNA at 900 ng/μl and the appropriate negative controls were included (see Fig. 5). Four hours after fertilization, corresponding to the 32–64 cell stage embryonic stage, embryos were fixed overnight at 4 °C in 4%PFA-MFSW (Millipore-Filtered Seawater buffer containing 4% paraformaldehyde). Then, samples were washed five times in PBS containing 0.1% Tween20 (PTw), dehydrated and permeabilized by increasing concentrations of methanol in PTw (25%, 50%, 75%, 100%), and stored at − 20 °C for at least 24 h. Samples were rehydrated by decreasing concentrations of methanol in PTw (75%, 50%, 25%), washed three times in PTw, followed by two washes in PBS-0.02% Triton X-100, then one time in PBS-0.2% Triton X-100 for 20 min, and again two times in PBS-0.02% Triton X-100. They were then blocked in PBS with 3% BSA for 3 h at RT and incubated overnight at 4 °C with a peroxidase-labeled anti-DIG antibody (Roche) diluted 1:1,500 in PBS with 3% BSA. Samples were then washed six to eight times for 15 min in PBS-0.02% Triton X-100 and incubated for 30 min at RT in Tyramide buffer (NaCl at 116.88 mg/ml and Boric Acid at 6.18 mg/ml in nuclease-free H_2_O; pH 8.5). Selected samples were incubated for 30 min at RT in development solution (Tyramide buffer, 0.0015% H_2_O_2_, NHS-Rhodamine at 4 μg/ml) to perform the tyramide signal amplification (TSA). All samples were washed six times in PTw and the last wash was left overnight at 4 °C. Nuclei were stained using Hoechst dye 33342 and samples were mounted in Fluoromount (Sigma-Aldrich).

### Nematocyst capsule staining

Mature nematocytes were counted by staining the poly-c-glutamate contents of their nematocyst capsules with DAPI as previously described^33^. Larvae were anesthetized in 4% MgCl_2_ in 50% MFSW/50% H_2_O for 30 min before fixation. Samples were fixed in nematocyst fix (10 mM EDTA, 4% PFA in PTw) at RT for 1 h, then washed three times in PTw containing 10 mM EDTA. Next, samples were incubated with DAPI at 1.43 μM in PBS1x overnight at 4 °C. This was followed by five to six washes in PTw with 10 mM EDTA, and samples were mounted in Fluoromount prior to imaging.

### Immunofluorescence

Larvae were anesthetized in 4% MgCl_2_ in 50% MFSW/50% H_2_O for 30 min prior to fixation. For immunofluorescence, samples were fixed at RT for 2 h in HEM buffer (0.1 M HEPES pH 6.9, 50 mM EGTA, 10 mM MgSO_4_) containing 0.02% Triton X-100% and 4% paraformaldehyde in MFSW, and then washed four times in PBS-0.3% Triton X-100. The last wash was left rocking overnight at 4ºC. Samples were then blocked in 3%BSA/5% goat serum in PBS-0.3% Triton X-100 for 3 h at RT and then incubated in primary antibody (rabbit anti-GLWamide^34^, kindly provided by N. Nakanishi) diluted to 1:200 in blocking solution overnight at 4 °C. This was followed by four washes in PBS-0.3% Triton X-100 before samples were blocked again in blocking solution for 1 h at RT, and then incubated with secondary antibody (goat anti-rabbit 556; Invitrogen) at a concentration of 1:500 in blocking solution for 1 h at RT. Animals were then washed four times for 15 min in PBS-0.3% Triton X-100 prior to nuclei staining using Hoechst dye 33342. Samples were mounted in Fluoromount prior to imaging.

For Supplementary Videos S1 and S2, embryos were electroporated with Dextran (Alexa Fluor 555; Invitrogen) at 1 mg/ml in Ficoll-400 15% MFSW and fixed 3 days post-electroporation in 4%PFA in PTw for 1 h at RT. Samples were washed in PBS-0.2% Triton X-100 for 15 min followed by several washes in PTw. To visualize nuclei, samples were stained using Hoechst dye 33342. Samples were mounted in Fluoromount prior to imaging.

### Confocal microscopy and cell counting

Images were acquired using a Zeiss LSM 710 confocal microscope. The same scanning parameters (i.e., magnification, laser intensity, and gain) were used for all conditions of each independent experiment. All supplementary videos as well as maximum intensity projections of z-stacks were prepared using ImageJ software^32^.

For DAPI-stained nematocysts, confocal z-stacks of ~ 10 μm focused on the larval surface were projected. Nematocysts were highlighted using custom thresholding, separated using a watershed filter and counted with the ‘Analyze Particles’ tool from ImageJ. For GLWamide^+^ neurons, confocal z-stacks of ~ 45 μm focused on the larval aboral region were projected and neuronal bodies were counted manually using the ImageJ ‘cell counting’ plugin.

### Graphs and statistical analyses

**Figure 6:**
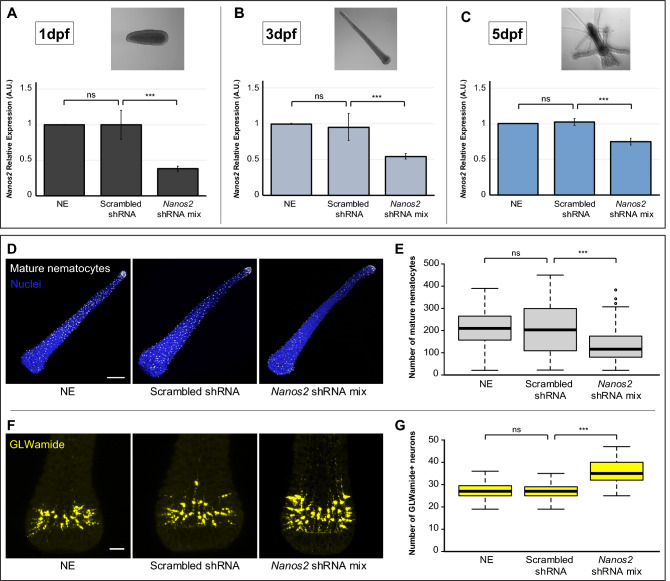
*Nanos2* knockdown quantification over time and phenotypic characterization of *Nanos2* knockdown larvae. (**A**–**C**) RT-qPCR results showing comparable expression levels of *Nanos2* mRNA in both controls, but a significant decrease (p value ≤ 0.01) of *Nanos2* mRNA expression levels when comparing *Nanos2* shRNA mixture-electroporated samples to the scrambled shRNA control at 1 dpf (**A**), 3 dpf (**B**), and 5 dpf (**C**). Bright field images of non-electroporated animals are shown to display the animals’ morphology at each timepoint of RNA extraction. In all cases, levels of *Nanos2* expression in scrambled shRNA and *Nanos2* shRNA mixture (mix) samples were quantified relative to the NE control. Bar heights represent mean values of three independent experiments and error bars show standard deviations. *ns* non-significant; ***p value ≤ 0.01. (**D**) Representative images of 3 dpf larvae showing mature nematocytes (white) on the larval surface for each of the three different conditions presented. Cell nuclei are stained in blue. Images were projected from confocal stacks of ~ 10 μm. (**E**) Box plot showing the number of mature nematocytes for each of the three conditions. Center lines show the medians; box limits indicate the 25th and 75th percentiles (first and third quartiles); whiskers extend 1.5 times the interquartile range from the 25th and 75th percentiles; outliers are represented by circles. For NE, n = 49; for scrambled shRNA, n = 53; for *Nanos2* shRNA mixture (mix), n = 65. (**F**) Representative images of 3 dpf larvae showing the GLWamide^+^ neurons (yellow) on the larval aboral region for each of the three different conditions. Images were projected from confocal stacks of ~ 45 μm. (**G**) Box plot showing the number of GLWamide^+^ neurons for each of the three conditions. Box plot characteristics as in (**B**). For NE, n = 72; for scrambled shRNA, n = 68; for *Nanos2* shRNA mixture, n = 75. Altogether, *Nanos2* knockdown yields a significant reduction (p value ≤ 0.01) of mature nematocytes and a significant increase (p value ≤ 0.01) of GLWamide^+^ neurons in the 3 dpf larvae. *Ns* non-significant; ***p value ≤ 0.01. Scale bars 100 μm in (**D**) and 25 μm in (**F**).

Box plots for Fig. 6 and Supplementary Figs. S4 were prepared using BoxPlotR^35^. All other graphs were prepared in Excel and assembled using Adobe Illustrator.

To assess RT-qPCR statistical significance, we used the delta-delta-Ct values to perform Shapiro–Wilk normality tests and two-tailed Student’s t tests. A Benjamini–Hochberg correction was applied to account for multiple comparisons. For statistics related to counts of nematocysts and GLWamide^+^ neurons (Fig. 6), Kolmogorov–Smirnov normality tests were used, and two-tailed Student’s t tests were performed. For tentacle number comparisons (Supplementary Fig. S4), the nonparametric Mann–Whitney U test was chosen since the results did not follow a normal distribution according to the Kolmogorov–Smirnov test, and significance between sample distributions could be appropriately assessed by data transformation into ranks. All statistical tests were performed at https://www.socscistatistics.com.

## Results

### Initial electroporation trials with Dextran

We first tested the efficiency of Dextran transfection into *H. symbiolongicarpus* one-cell stage embryos using different electroporation conditions. Dextrans are polysaccharides that can be synthetically labelled, and they have previously been used as long-term tracers in cnidarians^23,36^. The Dextran (Alexa Fluor 555; Invitrogen) used in our experiments fluoresces when excited with green light and has a similar molecular mass to shRNAs (10,000 Da and ~ 15,000 Da, respectively). We reasoned that electroporation trials with Dextran would help to visually demonstrate whether small molecules can be successfully transfected into *H. symbiolongicarpus* embryos and to test conditions to maximize the survivorship of embryos and overall transfection efficiency.

Our initial electroporation trials were performed with unfertilized eggs that were subsequently fertilized, but we found that fertilizing after electroporation led to almost no healthy embryos. We performed all subsequent trials with one-cell stage fertilized embryos, which resulted in high percentages of healthy, developing embryos. We tested six different electroporation conditions (conditions 1–6), modifying the voltage (volts—V), the number of pulses, and/or the pulse length (milliseconds—ms) in each trial (Supplementary Fig. S1). Electroporation trials were carried out as described in Fig. 2, but instead of shRNAs, Dextran was diluted in 15% Ficoll-400 MFSW at a concentration of 1 mg/ml. We defined successful electroporation conditions as those that yielded high percentages of Dextran^+^ larvae as well as high survival rates at 1 day post-fertilization (dpf). A non-electroporated (NE) control was added, where samples were soaked in Dextran diluted in 15% Ficoll-400 MFSW at the same concentration for ~ 3 min, the approximate length of an electroporation procedure, to allow appropriate comparisons. We found that the 1 dpf larvae in the NE controls were almost non-fluorescent and the survival rate was near 100%, as expected (Supplementary Fig. S1). Of the six electroporation conditions tested, conditions 1–3 showed the highest percentages of Dextran^+^ 1 dpf larvae (Supplementary Fig. S1). However, condition 3 (25 V, 1 pulse, 25 ms) gave the highest survivorship, with a 1 dpf survival rate of 85% (Supplementary Fig. S1). We then fixed the larvae from condition 3 and the NE control at the 3 dpf stage, and used confocal microscopy to confirm that the electroporation parameters from condition 3 had successfully delivered Dextran inside the embryos and that the fluorescence was distributed among most cells throughout development (Supplementary Videos S1, S2). The success of electroporation condition 3 in delivering Dextran gave us confidence to begin testing shRNA transfection in *H. symbiolongicarpus* embryos.

### Optimization of shRNA electroporation

Following our Dextran trials, we tested several electroporation conditions to assess the success in delivering shRNAs into one-cell stage embryos, and to determine the extent of shRNA-mediated gene knockdown in *H. symbiolongicarpus*. For these trials, we chose to target a gene with obvious phenotypic characteristics upon knockdown. We thus targeted the *eGFP* gene in the offspring of a cross between eggs produced by a wildtype female line (295–8) and sperm from a transgenic *Eef1alpha* > *eGFP* male line (354–3)^10^. The transgenic line was created via CRISPR/Cas9-mediated *eGFP* gene knockin to the endogenous housekeeping gene *Eef1alpha* (eukaryotic elongation factor 1 alpha) locus. The line is heterozygous for the transgene and expresses it in all cells^10^. In crosses between the transgenic male and the wildtype female, we consistently obtained approximately 50% eGFP^+^ larvae at 1 dpf (Fig. 3A), as expected by Mendelian inheritance.

We designed three different shRNAs targeting various regions of the *eGFP* mRNA (see “Methods”). We decided to exclude all sequences that had 16 complementary nucleotides or more with non-target genes to minimize the risk of off-target effects^21^. Based on previous studies in *N. vectensis*^23,24^, we hypothesized that a concentration of 300 ng/μl for each shRNA should be effective in inducing the knockdown of most genes for several days in *H. symbiolongicarpus*. We decided to test a strategy of electroporating a mixture of three shRNAs at a time, each at a concentration of 300 ng/μl per shRNA (total concentration of 900 ng/µl), with the goal of achieving high knockdown levels of the targeted gene. We reasoned that testing three shRNAs at once would be effective even if one shRNA was less active than the others or if the activity varied among shRNAs, and that this strategy would be a good starting point for screening the knockdown of multiple genes per spawning event, albeit with a slightly higher risk of off-targeting.

We carried out electroporation trials as detailed in Fig. 2 under varying electroporation parameters and experimental conditions (Supplementary Fig. S2). *eGFP* shRNA mixtures were diluted in 15% Ficoll-400 MFSW to a concentration of 300 ng/μl per shRNA (900 ng/μl total). In all cases, we assessed survivorship and the number of eGFP^+^ larvae at 1 dpf (Supplementary Fig. S2). To evaluate any potential deleterious effects from high concentrations of shRNAs, we also tested two different control electroporation conditions where nuclease-free H_2_O was added instead of shRNAs (‘No shRNA’ controls). We observed that the survivorship levels in the ‘No shRNA’ controls were comparable to the conditions where the *eGFP* shRNA mixture was added (Supplementary Fig. S2), indicating that shRNA electroporation at a final concentration of 900 ng/μl was not toxic and did not affect survivorship levels of the animals. We also observed that electroporation of the *eGFP* shRNA mixture was successful in yielding lower percentages of eGFP^+^ larvae than in controls at 1 dpf under all conditions tested (Supplementary Fig. S2). This result strongly suggested that *eGFP* knockdown could be accomplished through shRNA electroporation of *H. symbiolongicarpus* embryos. In agreement with the Dextran trials, the optimal electroporation condition for shRNA transfection, based on highest survivorship (~ 85%) and lowest percentage of eGFP^+^ larvae (~ 2% of ~ 50% expected), was 25 V, 1 pulse, 25 ms (Condition A, Supplementary Fig. S2). Thus, we used these electroporation parameters for all subsequent shRNA experiments.

We then visually screened whether electroporation of the *eGFP* shRNA mixture yielded a stronger reduction in eGFP fluorescence levels than that of each of the three shRNAs (*eGFP* shRNAs 1–3) when electroporated separately. In one set of experiments, each shRNA was tested at the concentration they have in the mixture (i.e., 300 ng/μl each) and compared to the mixture (total concentration of 900 ng/µl). In another set of experiments, each shRNA was tested at the same final concentration as the mixture (i.e., 900 ng/µl each) and compared to the mixture (total concentration of 900 ng/µl). We included a NE control and a scrambled shRNA control for each experiment (Fig. 3B). The scrambled shRNA was designed from a randomized sequence that was verified not to target any gene in the *H. symbiolongicarpus* genome. Thus, the scrambled shRNA served as a negative shRNA control when added at the highest concentration of the experiment (900 ng/μl). We acquired fluorescent images of 1 dpf larvae for each condition. In all experiments, the NE and scrambled shRNA controls showed the expected percentage of highly fluorescent 1 dpf larvae (~ 50%; Fig. 3B). All electroporation conditions yielded a 75–97% survival rate at 1 dpf (Supplementary Table S2). For the experiments where each individual shRNA was tested at a concentration of 300 ng/µl, *eGFP* shRNA 1 produced the most obvious reduction in eGFP fluorescence of the three individual shRNAs, but the *eGFP* shRNA mixture appeared to show the strongest fluorescence reduction of all conditions when screened visually. For the experiments where each individual shRNA was tested at a concentration of 900 ng/µl, we visually screened the fluorescence levels and observed that shRNAs 1 and 2 displayed virtually the same low level of fluorescence as the mixture, whereas embryos electroporated with shRNA 3 exhibited a slightly higher overall fluorescence level (Fig. 3B). Importantly, the fluorescence reduction upon any *eGFP* shRNA electroporation condition was evident throughout all tissues of each larva (Fig. 3B), suggesting that low levels of mosaicism occur following our method.

To verify that the reduction in fluorescence levels was due to a reduction in *eGFP* transcript levels and to analyze the degree of gene-specific knockdown, we performed RT-qPCR analyses on 1 dpf larvae (Fig. 3C,D). We first compared the *eGFP* mRNA levels between the NE and scrambled shRNA controls. As expected, the difference in the *eGFP* mRNA levels was not significant between the controls (Fig. 3C). We then measured the *eGFP* mRNA levels for all experimental conditions relative to the scrambled shRNA control (Fig. 3D). For all conditions, we found significant reductions in *eGFP* mRNA expression (Student’s t test; p value ≤ 0.01), with the *eGFP* shRNA mixture and shRNA 1 giving the most dramatic knockdowns (92% and 85% transcript reduction, respectively) in the set of experiments where each individual shRNA was tested at the lower 300 ng/µl concentration. When we compared each shRNA at 300 ng/µl concentration to the *eGFP* shRNA mixture, the mRNA levels in the mixture were significantly lower than in two of the individual *eGFP* shRNAs alone (shRNA 2 and 3), although the difference between *eGFP* shRNA 1 and the mixture was not significant, indicating that *eGFP* shRNA1 yielded the strongest knockdown of the three individual shRNAs (Fig. 3D). In the set of experiments where the individual shRNAs were each at the higher concentration of 900 ng/µl, all shRNAs gave a knockdown similar to the mixture condition and the observed differences were not statistically significant (Fig. 3D). Thus, there was an effect of shRNA concentration on targeted transcript levels, with the higher concentration of 900 ng/µl being more effective in reducing *eGFP* transcript levels for all individual shRNAs tested. Together, these results show that the qualitative reduction in eGFP fluorescence levels we observed following *eGFP* shRNA electroporation represent successful gene-specific knockdowns. The results also show that the *eGFP* shRNA mixture strategy produces a knockdown that is as effective and dramatic as the most efficient individual shRNA at the highest concentration. Given these results, we opted to use the shRNA mixture strategy for all subsequent experiments.

### Characterization of *eGFP* knockdown over time

To ensure that shRNA electroporation would not have harmful consequences on the developing animals in the long-term, we decided to follow embryos subjected to electroporation with the *eGFP* shRNA mixture through an extended time course. We compared a NE control sample to an *eGFP* shRNA mixture-electroporated sample for 28 dpf. We examined survivorship during the first 3 days of development in both samples and observed that, by 3 dpf, knockdown animals showed a 73.8% survival rate, as compared to a survival rate of 90.2% in the NE controls (Supplementary Table S2). This 16.4% difference in survivorship between samples is likely explained by inherent electroporation-induced damage (Supplementary Fig. S2). We determined that the highest embryo mortality occurred during the first 2 days of development in both NE and electroporated animals. The surviving knockdown larvae at each developmental stage did not exhibit any noticeable negative appearance when compared to the NE sample, and all surviving individuals became fully developed 3 dpf larvae (Supplementary Fig. S3). Both NE and 3 dpf knockdown larvae were competent to metamorphose into primary polyps upon CsCl stimulation (see “Methods”). The two samples had very similar metamorphosis rates (77.0% NE and 78.2% *eGFP* knockdown; Supplementary Table S2), indicating no negative effect of shRNA electroporation on metamorphosis. Mortality after metamorphosis was virtually absent in both cases. To ensure the survivorship of the metamorphosed polyps, we fed them smashed brine shrimp throughout the length of the experiment. We first fed the young primary polyps at 5 dpf, the stage by which their mouths are fully formed, and subsequent feedings were done every other day. All polyps from both samples could be successfully fed and steadily grew to form polyp colonies (Supplementary Fig. S3). These results indicate that *eGFP* shRNA mixture-electroporated embryos are capable of fully developing into 3 dpf larvae, metamorphosing into primary polyps, feeding, and growing to eventually form a colony.

We were also interested in documenting the eGFP fluorescence dynamics over time in *eGFP* knockdown animals compared to the NE control, where the fluorescence level corresponds to the amount of eGFP protein inside the cells. To evaluate this, we obtained fluorescent images of both samples at different timepoints throughout the experiment and quantified fluorescence levels to understand the phenotype dynamics over time (Fig. 4A,B). The fluorescence levels in the NE animals increased during the first 2 days of embryonic development, remained steady by 3 dpf, and increased again reaching an upper threshold (fluorescence saturation) after metamorphosis was achieved (4 dpf). Fluorescence then remained relatively stable until the end of the experiment (Fig. 4B).

In contrast, in *eGFP* knockdown animals, eGFP fluorescence was virtually absent throughout development and in 4–5 dpf primary polyps. By 6 dpf we observed that fluorescence in the knockdown polyps started to return (Fig. 4A,B), suggesting that the effectiveness of the shRNA-induced knockdown started to diminish at this stage. The fluorescence levels kept increasing over time, although it took up to 28 dpf for the *eGFP* shRNA mixture-electroporated animals to reach equivalent fluorescence levels to the NE controls (Fig. 4A,B). These results suggest that electroporation of shRNAs that yield a high reduction of transcript levels such as the one we showed for *eGFP* (Fig. 3D), can maintain an effective knockdown for up to 6 days post-fertilization (2 days post-metamorphosis). They also suggest that *eGFP* shRNA electroporation-induced knockdown animals need several weeks to fully recover normal eGFP protein levels and thus reach equivalent fluorescence levels as the NE control.

### Visual confirmation of successful shRNA transfection via electroporation

To have visual confirmation that our optimized electroporation parameters (1 pulse, 25 V, 25 ms length, 2 mm-gap cuvettes) successfully delivered shRNAs inside one-cell stage embryos of *H. symbiolongicarpus*, we synthesized scrambled shRNA labeled with digoxigenin (Dig-shRNA) and electroporated embryos with this construct at 900 ng/μl in 15% Ficoll-400 MFSW. We chose to use scrambled shRNA in this experiment to avoid any interference of gene expression upon electroporation of the Dig-shRNA construct. Appropriate controls were performed (described in Fig. 5A–C legend). By developing a tyramide signal amplification (TSA) reaction in fixed 4 hpf embryos (32–64 cell stages), we observed a strong fluorescence signal that indicated the presence of Dig-shRNA inside the embryos’ cells (Fig. 5D; Supplementary Videos S3, S4). Every cell within each embryo (n = 28 embryos) that we analyzed showed fluorescence, although some cells displayed a stronger signal than others. Labeling shRNAs with digoxigenin proved to be a successful approach to trace the shRNAs inside the embryos upon electroporation, and is inexpensive when compared to other commercial labeling kits^37^. This visual confirmation indicates that shRNAs are successfully delivered inside one-cell stage embryos with our optimized electroporation parameters, and suggests that shRNAs are then stochastically distributed among cells throughout embryonic development.

### Endogenous gene knockdown through shRNA electroporation

To test whether our method could be used to knock down endogenous genes in *H. symbiolongicarpus*, we targeted the *Nanos2* gene, which is known to be essential for balancing the numbers of neurons and nematocytes (stinging cells) in the sister species *H. echinata*^11^. *Nanos2* knockdown was previously achieved through microinjection of a translation blocking morpholino. We designed three different shRNAs targeting different regions of *Nanos2* mRNA and electroporated one cell-stage embryos with a *Nanos2* shRNA mixture (300 ng/μl per shRNA in 15% Ficoll-400 MFSW, total concentration 900 ng/µl). Average survivorship in *Nanos2* knockdown 1 dpf larvae was ~ 80% (Supplementary Table S2) and all surviving larvae developed successfully and metamorphosed into polyps. We carried out RT-qPCR analyses using mRNA samples collected at different timepoints (1, 3, and 5 dpf) to evaluate *Nanos2* knockdown efficiency over time. The *Nanos2* relative expression was reduced by 62% at 1 dpf, 45% by 3 dpf, and only 26% at the post-metamorphic stage of 5 dpf (Fig. 6A–C). In all cases, however, *Nanos2* shRNA mixture-electroporated samples showed a statistically significant reduction in *Nanos2* mRNA levels when compared to scrambled shRNA controls. Our results strongly suggest a successful knockdown of *Nanos2* can be achieved via shRNA electroporation of fertilized eggs, lasting throughout embryonic development.

We then performed phenotypic analyses in our knockdown animals by counting the number of mature nematocytes and GLWamide^+^ neurons in 3 dpf larvae, as well as the number of tentacles in 5 dpf primary polyps. We observed that *Nanos2* knockdown larvae displayed a significantly lower number of mature nematocytes, as well as a significantly higher number of GLWamide^+^ neurons than scrambled shRNA controls (Fig. 6D**–**G). We also detected a significant decrease in tentacle numbers in *Nanos2* knockdown 5 dpf primary polyps compared to scrambled shRNA control polyps (Supplementary Fig. S4). Additionally, we observed that most of the tentacles in the knockdown polyps were shorter than in the controls. Altogether, these results show that we successfully obtained the *Nanos2* knockdown phenotype previously described^11^ using our shRNA mixture electroporation strategy.

To confirm the efficiency and reproducibility of our method, we attempted to knock down other *H. symbiolongicarpus* endogenous genes. Because *H. symbiolongicarpus* is a useful model for stem cell research, we chose to target the genes *GNL2* and *GNL3,* which are interesting candidates for understanding stem cell dynamics in our animal due to their conserved function in ribosomal biosynthesis and to their high expression in proliferating and tumorigenic cells^38^. We designed three different shRNAs targeting different regions of these genes, and electroporated one cell-stage embryos with shRNA mixtures for each (300 ng/μl per shRNA in 15% Ficoll-400 MFSW, total concentration 900 ng/µl). We then carried out RT-qPCR analyses using mRNA samples collected at 1 dpf to assess the efficiency of the knockdown. Both *GNL2* and *GNL3* shRNA mix-electroporated samples showed a reduction in their transcript levels (47% and 72% reduction, respectively), which were statistically significant when compared to scrambled shRNA controls (Supplementary Fig. S5), strongly suggesting a successful knockdown of these genes can be achieved using our method. We did not attempt to perform phenotypic analyses of these genes since the phenotype is unknown as they have not been previously studied in cnidarians and will be the subject of a future study.

## Discussion

By studying a larger cross-section of research organisms, we deepen our understanding of biological processes in the broader context of evolution and contribute to our knowledge of basic biological mechanisms common to all animals^39,40^. Thus, the ability to perform gene function analyses in a wide range of organisms across the animal tree is essential for understanding the biological functions of genes, and how these functions have changed throughout evolutionary time. With the advent of gene editing tools, the ability to perform functional genomic analyses in a diversity of species has become remarkably accessible^41,42^. RNAi-based tools such as shRNAs, however, still remain a popular choice within the widening array of technologies available to disrupt gene function, since they allow the study of gene function in a faster and easier way than the creation and maintenance of a knockout line^43^. Moreover, the temporary reduction of gene expression instead of the complete disruption of a gene avoids the problem of embryonic lethality given by the knockout of some genes that are crucial for development or survival, thus allowing the study of their function. Deciding which tool to choose for a particular experiment will depend on the biological question a researcher aims to answer, the gene targeted, and the life stage of the organism that one is targeting^43^.

In this study, we have developed a method to temporarily induce gene knockdown in *H. symbiolongicarpus* embryos via shRNA electroporation of fertilized eggs. This method has several advantages, and electroporation represents an attractive alternative to other delivery methods such as microinjection or soaking. The design of shRNAs is straightforward (Supplementary Info S2) and their synthesis is inexpensive compared to other antisense molecules such as morpholinos^44^. Moreover, shRNAs are advantageous activators of the endogenous RNAi pathway since they retain a relatively low rate of degradation and turnover^17^. Another important benefit lies in the large number of embryos that can be rapidly and efficiently transfected at a single time via electroporation, as compared to microinjection, allowing for a higher number of experimental conditions per spawning event. We have demonstrated that using an shRNA mixture strategy is a valid and time-saving starting point as it allows for screening the knockdowns of several genes from a single spawning event, rather than having to test several shRNAs individually. The ideal experimental design to perform gene function analyses, however, would consist of the combination of two different gene disruption strategies (e.g. knockout and knockdown), or the use of two non-overlapping shRNAs individually with a reproducible phenotype, to minimize and account for any potential off-target effects of pooling shRNAs.

Our method has allowed us to recapitulate a morpholino microinjection-induced knockdown phenotype of the endogenous gene *Nanos2* that was previously shown in *H. echinata*^11^. The penetrance of the phenotype via morpholino microinjection was comparable to that of our shRNA mixture electroporation, as the two methods gave very similar results (37.5% vs 35.2% decrease in mature nematocytes, respectively, and 36% vs 32.5% increase in neurons, respectively). A recent study in *H. symbiolongicarpus* showed that co-microinjection of an shRNA at 500 ng/µl targeting the *GFP* gene, and a plasmid that ectopically expressed GFP into fertilized embryos, led to the absence of GFP fluorescence in 100% of the animals at 3 dpf and its recovery by 10 dpf^25^. Our *eGFP* shRNA mixture electroporation strategy allowed us to obtain a similar phenotypic penetrance as the one obtained by shRNA microinjection, based on almost total absence of eGFP^+^ larvae at 1 dpf (Supplementary Fig. S2), and the slow recovery of eGFP fluorescence (Fig. 4A,B). Therefore, based on the comparable phenotypic penetrance of the different methods discussed, we would encourage the use of shRNA electroporation for targeted gene knockdown as it allows for the transfection of a much higher number of embryos in a shorter amount of time than shRNA or morpholino microinjection. The co-transfection of multiple biomolecules into eggs, however, has not yet been attempted and might prove challenging via electroporation, in which case microinjection may be a better approach.

shRNA electroporation of larval and post-metamorphic stages remains to be established in *H. symbiolongicarpus*. Based on our RT-qPCR data of *Nanos2* knockdown animals at different life cycle stages (Fig. 6A–C), and on our eGFP fluorescence quantification over time of *eGFP* knockdown animals (Fig. 4A,B), however, we estimate that electroporating fertilized eggs with shRNAs can be used at the very least to assess the knockdown of genes throughout development. Effective knockdowns using our method might nonetheless present limitations starting at post-metamorphic stages. For instance, *Nanos2* knockdown levels at 5 dpf (Fig. 6C) might not be strong enough to produce clear phenotypes. shRNAs electroporated into fertilized eggs have not yet been shown to affect the function of any endogenous gene after metamorphosis in *H. symbiolongicarpus*, nor to substantially lower the expression levels at post-metamorphic timepoints. Metamorphosis represents a radical change in the life cycle of *Hydractinia*, encompassing cell rearrangements and apoptosis^45,46^ as well as high levels of cell proliferation^47^. Throughout this dramatic process, most transfected shRNAs in the one-cell stage embryo are likely diluted and/or degraded, making it difficult to maintain their biological activity, and thus a strong knockdown effect, after metamorphosis takes place.

Based on our studies, we believe that a high concentration of shRNAs (i.e. 900 ng/µl) should provide a clear knockdown of a targeted gene that should last at least throughout embryogenesis in *H. symbiolongicarpus*. However, the efficiency of our method in reducing targeted transcript levels has shown to be variable among genes, with reductions of 45–75% at 1 dpf for endogenous genes (see above), and > 90% reduction for the *eGFP* exogenous gene. Due to the variable knockdown levels achieved when targeting endogenous genes, gene function analyses might not always be easy to assess. Moreover, the duration of shRNA-induced knockdown may have to be tested specifically for each targeted gene. The length of a particular knockdown likely depends on the efficiency of the initial mRNA disruption. If allelic variation exists in targeted genes, it would also be helpful to target regions of endogenous genes with low polymorphism for shRNA design, as this will help to ensure efficient binding of shRNAs to their target. Thus, achieving the most efficient and durable shRNA electroporation-induced knockdown will require designing and finding the best shRNA(s) and their optimal concentration for each target gene.

Electroporation of shRNAs into unfertilized eggs has been recently described for the anthozoan cnidarian *N. vectensis*^24^. The few differences between our method and the one presented for *N. vectensis*, such as electroporating fertilized eggs in *H. symbiolongicarpus* compared to unfertilized eggs in *N. vectensis*, are due to the distinct biology of the two species. *H. symbiolongicarpus* diverged from *N. vectensis* ~ 600 million years ago^48^ so it is unsurprising that their biology is quite different. One key difference is that *H. symbiolongicarpus* is a fully marine species while *N. vectensis* is estuarine (cultured in 1/3 strength seawater), thus it cannot be taken for granted that shRNA electroporation will behave the same in these two cnidarian species. The community of cnidarian researchers is rapidly broadening and becoming more interdisciplinary^49^, highlighting the need to develop new methods to study gene function in multiple species. Our comprehensive testing and success in inducing gene-specific knockdowns via shRNA electroporation of fertilized eggs in *H. symbiolongicarpus*, therefore, represents a methodological springboard for researchers studying gene function throughout embryonic development in a variety of cnidarian species. We hope that our detailed methodology will inspire researchers who work with diverse research organisms, especially marine invertebrates, to use similar strategies to knock down genes of interest.

## Supplementary information

Supplementary file1

Supplementary file2

Supplementary file3

Supplementary file4

Supplementary file5

Supplementary file6

Supplementary file7
