# Publisher Correction: Gene knockdown via electroporation of short hairpin RNAs in embryos of the marine hydroid *Hydractinia symbiolongicarpus*

**DOI:** 10.1038/s41598-020-73062-8

**Published:** 2020-10-08

**Authors:** Gonzalo Quiroga-Artigas, Alexandrea Duscher, Katelyn Lundquist, Justin Waletich, Christine E. Schnitzler

**Affiliations:** 1 Whitney Laboratory for Marine Bioscience, University of Florida, St. Augustine, FL 32080 USA; 2 Department of Biology, University of Florida, Gainesville, FL USA

Correction to: *Scientific Reports* 10.1038/s41598-020-69489-8, published online 30 July 2020

The original PDF version of this Article contained an error in Figure 3A, where there was a missing component for the schematic depiction of the Wild Type Colony due to a technical error. This has now been corrected in the PDF; the HTML version of the paper was correct from the time of publication.

