# Supplementary material for: Gene knockdown via electroporation of short hairpin RNAs in embryos of the marine hydroid Hydractinia symbiolongicarpus
Source: Sci Rep. 2020 Jul 30;10:12806. doi: 10.1038/s41598-020-69489-8 (PMC7393174; doi:10.1038/s41598-020-69489-8)
Supplement: Supplementary file 7 — Supplementary file7 [file 41598_2020_69489_MOESM7_ESM.docx]

Supplementary Information

Gene knockdown via electroporation of short hairpin RNAs in embryos of the marine hydroid *Hydractinia symbiolongicarpus*

Gonzalo Quiroga-Artigas^1,*^, Alexandrea Duscher^1^, Katelyn Lundquist^1^, Justin Waletich^1^, Christine E. Schnitzler^1,2,*^

^1^ Whitney Laboratory for Marine Bioscience, University of Florida, St. Augustine, FL, USA 32080

^2^ Department of Biology, University of Florida, Gainesville, FL, USA

* Corresponding authors: [gonzalo.artigas@whitney.ufl.edu](mailto:mailto:%20gonzalo.artigas@whitney.ufl.edu), [christine.schnitzler@whitney.ufl.edu](mailto:christine.schnitzler@whitney.ufl.edu)


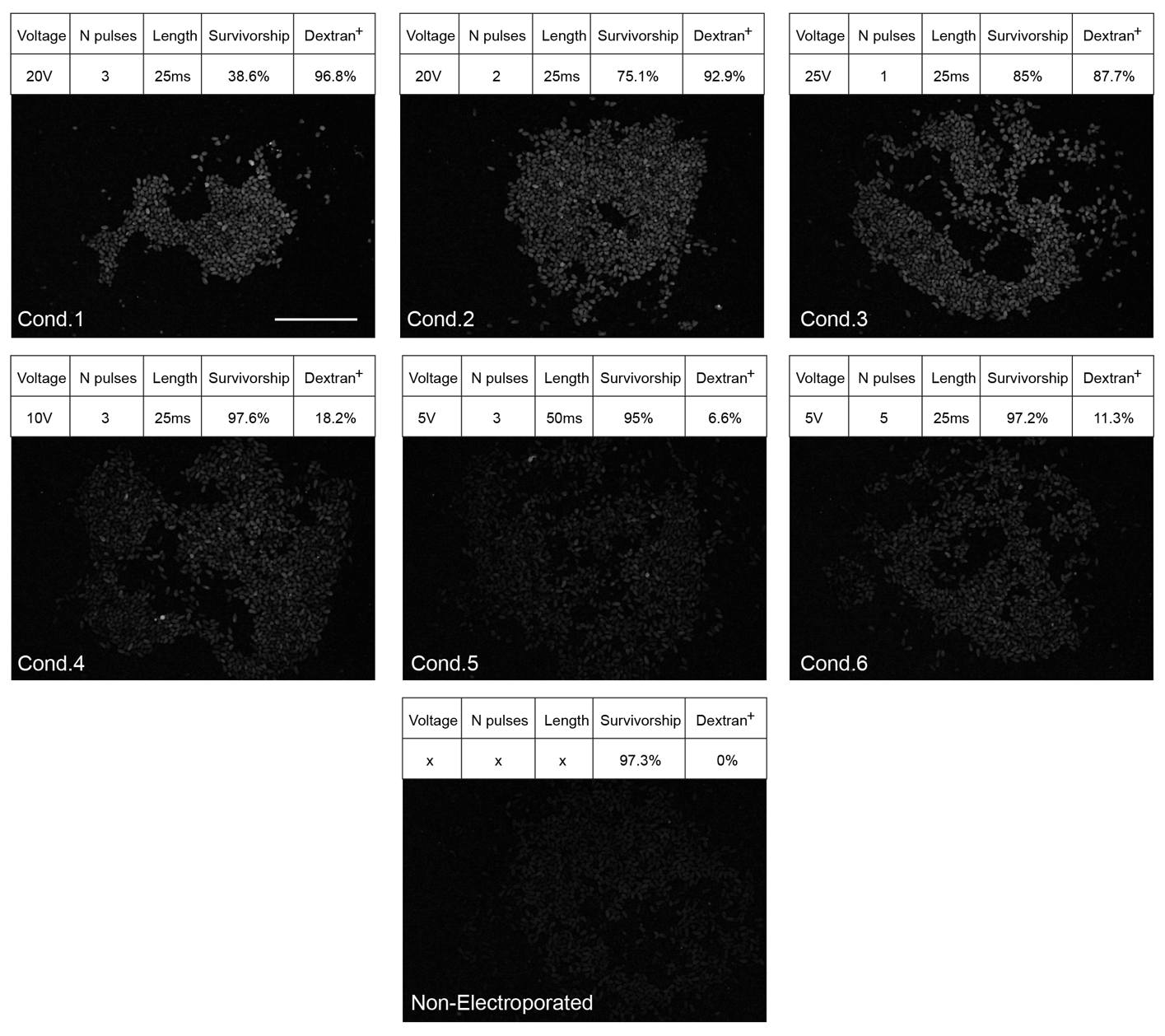


Supplementary Figure S1. **Dextran embryo transfection under different electroporation conditions**. Conditions (cond.) 1-6 account for different electroporation parameters (voltage, number of pulses, and pulse length) tested to deliver Dextran inside one-cell stage embryos. Images show 1 dpf larvae displaying different levels of Dextran fluorescence (grey), which depended on how successful the delivery was from each condition. Note the almost complete lack of fluorescence in the non-electroporated control, indicating a lack of Dextran delivery inside the embryos. Percentages of survivorship and Dextran^+^ embryos at 1 dpf for each condition are also shown. Based on the number of Dextran^+^ embryos and the survival rate, condition 3 gave the most promising electroporation parameters for Dextran delivery into *H. symbiolongicarpus* embryos. All images come from a single experiment on the same batch of embryos. Scale bar = 500μm.


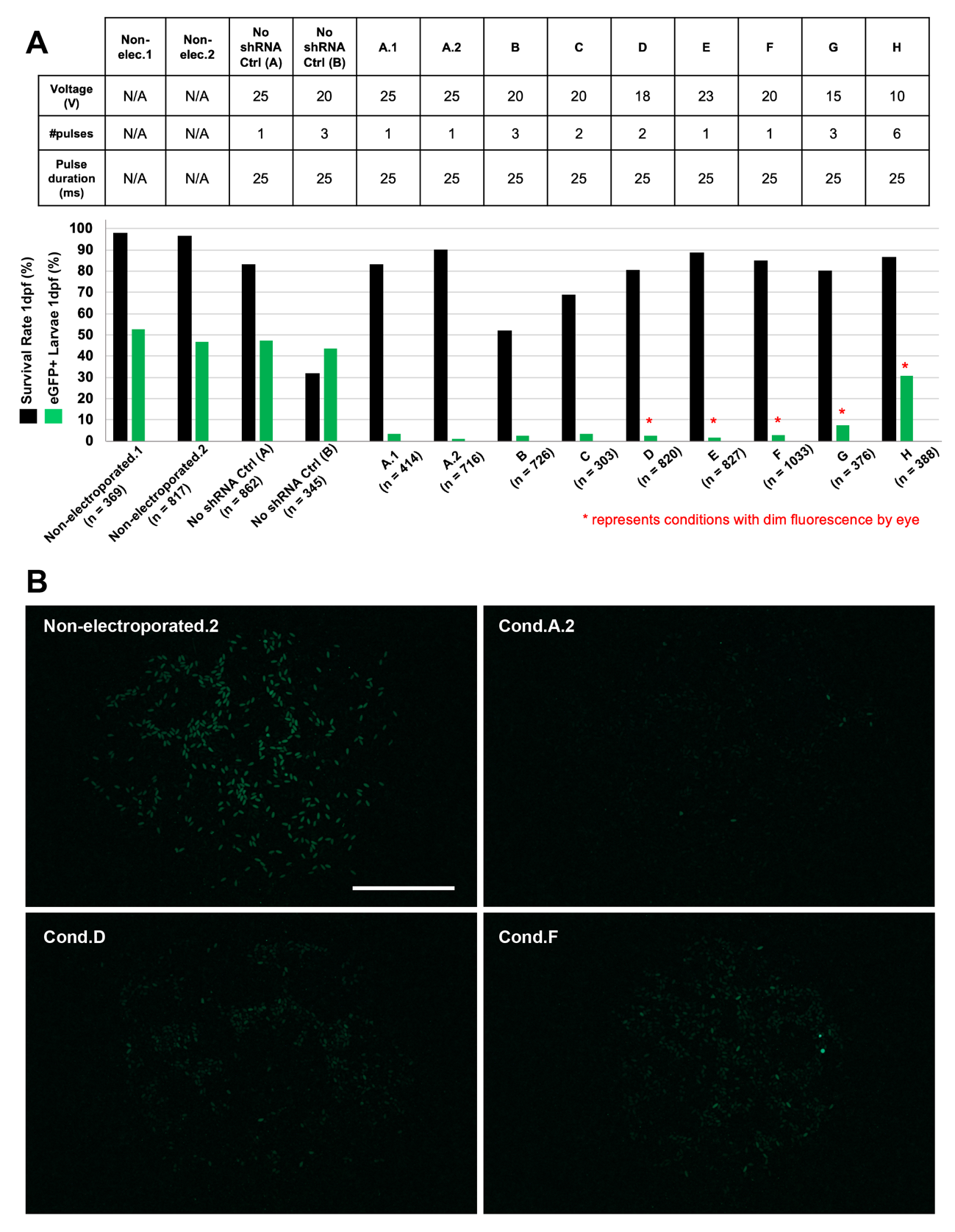


Supplementary Figure S2. **Survivorship and eGFP^+^ larvae percentage for different electroporation conditions**. (**A**) Double-bar graph showing the survival rate and percentage of eGFP^+^ larvae at 1 dpf after different electroporation conditions were used on fertilized eggs. The table above the graph containing the names of each condition and the electroporation parameters used corresponds vertically with the X axis of the graph. The names of the conditions that were repeated more than once are followed by a dot and a number, indicating the number of experiments where they were used. The number of electroporated embryos is shown for each condition at the bottom of the graph. Conditions A-H contained *eGFP* shRNA mixture in the electroporation solution. Note how the non-electroporated and the no shRNA controls present ~50% eGFP^+^ larvae by 1 dpf, as expected by Mendelian inheritance, whereas conditions A-H always show lower percentages. Conditions presenting dim fluorescence seen by eye (D-H) are marked with a red asterisk and represent those for which dimly fluorescent larvae could be observed under a stereoscope, although this dim fluorescence could not be captured by the ImageJ software following our counting protocol (see Methods). (**B**) Fluorescence images of 1 dpf larvae for the conditions (cond.) presented. Notice the dimly fluorescent larvae in conditions D and F, likely indicating a less efficient delivery of *eGFP* shRNA mixture inside the embryos than in condition A. Based on the high survivorship and the low percentage of eGFP^+^ larvae observed, condition A gave the most successful electroporation parameters for shRNA delivery into *H. symbiolongicarpus* embryos. Scale bar = 500μm.


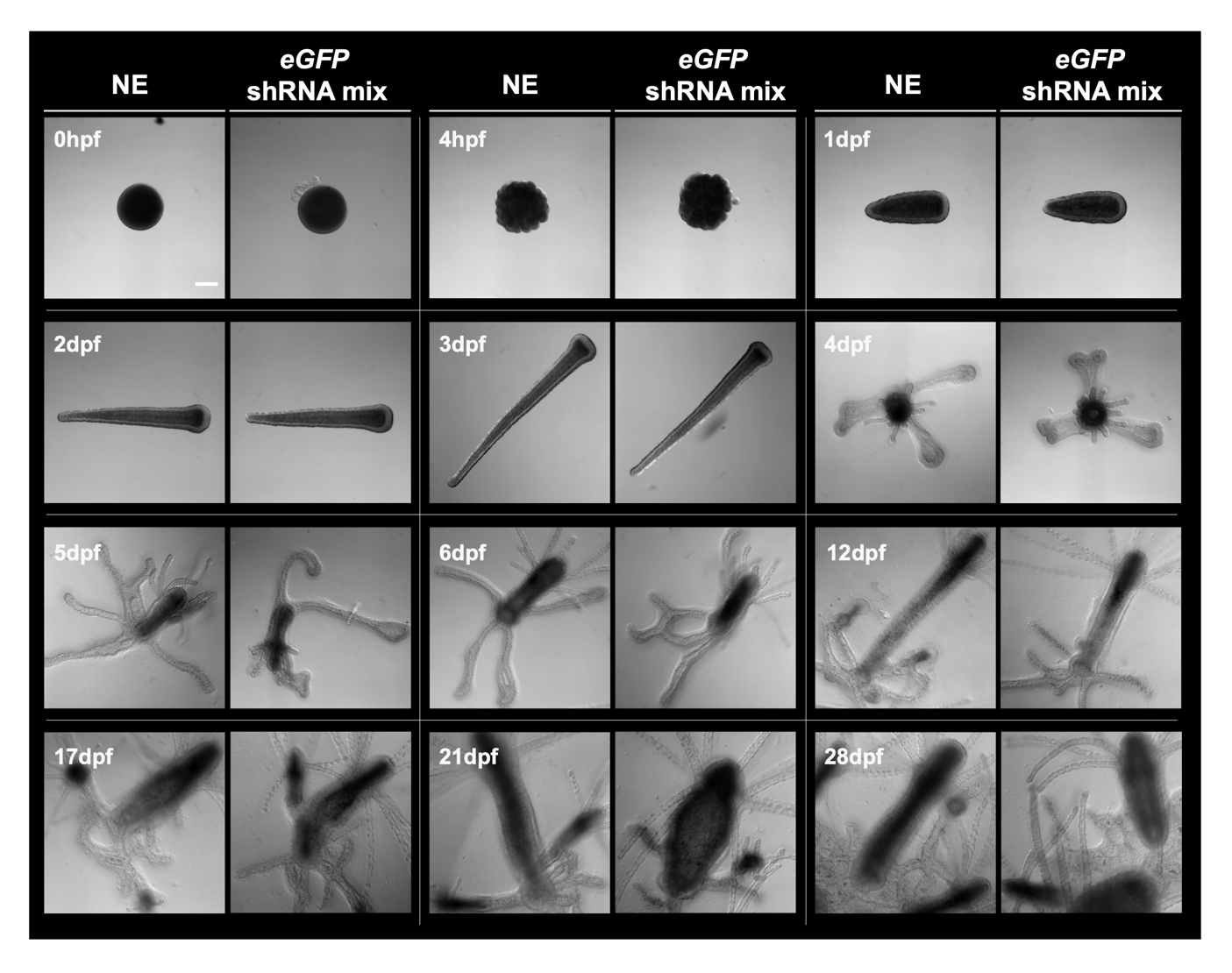


Supplementary Figure S3. **Morphology of *H. symbiolongicarpus* embryos and polyps is not negatively affected by shRNA electroporation**. Representative bright field images of embryonic and polyp stages are shown for each selected timepoint and condition. These bright field images correspond to the fluorescence images shown in Figure 4A. Scale bar = 100μm.

Supplementary Figure S4. **Tentacle number reduction in *Nanos2* knockdown animals**. (**A**) Representative images of 5 dpf primary polyps (2 days post-metamorphosis) for each of the three different conditions described. (**B**) Box plot showing the number of tentacles for each of the three displayed conditions. Center lines show the medians; box limits indicate the 25th and 75th percentiles (first and third quartiles); whiskers extend 1.5 times the interquartile range from the 25th and 75th percentiles; outliers are represented by circles. For NE, n = 201; for scrambled shRNA, n = 200; for *Nanos2* shRNA mixture (mix), n = 200. *Nanos2* knockdown yields a significant reduction (p-value ≤0.01) in the number of primary polyp tentacles. ns = non-significant; *** = p-value ≤0.01. Scale bar = 250μm.

Supplementary Figure S5. ***GNL2* and *GNL3* knockdown quantification**. (**A**) RT-qPCR assessing *GNL2* mRNA expression levels for different conditions performed using 1dpf larvae samples. The graph shows equivalent expression levels of *GNL2* mRNA in both controls, but a significant decrease (p-value ≤0.01) of *GNL2* mRNA expression levels when comparing *GNL2* shRNA mixture-electroporated sample to the scrambled shRNA control. Scrambled shRNA and *GNL2* shRNA mixture (mix) expression levels of *GNL2* were quantified relative to the NE control. (**B**) RT-qPCR assessing *GNL3* mRNA expression levels for different conditions carried out using 1dpf larvae samples. The graph shows comparable expression levels of *GNL3* mRNA in both controls, although a significant reduction (p-value ≤0.01) of *GNL3* mRNA expression levels is observed when comparing *GNL3* shRNA mixture-electroporated sample to the scrambled shRNA control. Scrambled shRNA and *GNL3* shRNA mixture expression levels of *GNL3* were quantified relative to the NE control. Bar heights represent mean values of at least three independent experiments and error bars show standard deviations. ns = non-significant; *** = p-value ≤0.01.

Supplementary Video S1. Representative 3 dpf larva from a non-electroporated control where one-cell stage embryos were soaked in 1mg/ml Dextran in 15% Ficoll-400 MFSW for ~3min, the approximate length of an electroporation procedure. The video shows a sequence of images taken from the surface of the larva to a mid-region where the endoderm is visible. This movie was generated at a frame rate of 2 fps in ImageJ from a 20μm confocal z-stack. Nuclei (blue), Dextran (red).

Supplementary Video S2. Representative 3 dpf larva from an experiment where one-cell stage embryos were electroporated with 1mg/ml Dextran in 15% Ficoll-400 MFSW. The video shows a sequence of images taken from the surface of the larva to a mid-region where the endoderm is visible. This movie was generated at a frame rate of 2 fps in ImageJ from a 30μm confocal z-stack. Nuclei (blue), Dextran (red).

Supplementary Video S3. Representative 4 hpf embryo from a non-electroporated control where one-cell stage embryos were soaked in 900 ng/μl Digoxigenin-labeled scrambled shRNA in 15% Ficoll-400 MFSW for ~3min, the approximate length of an electroporation procedure. Prior to imaging, the 4 hpf embryos were fixed, then incubated with peroxidase-labeled anti-DIG antibody, then tyramide signal amplification with rhodamine-tyramide was carried out (see Methods). The video shows a sequence of images taken from the surface of the embryo to a mid-region. This movie was generated at a frame rate of 2 fps in ImageJ from a 15μm confocal z-stack. Nuclei (blue), rhodamine-tyramide (red).

Supplementary Video S4. Representative 4 hpf embryo from an experiment where one-cell stage embryos were electroporated with 900 ng/μl Digoxigenin-labeled scrambled shRNA in 15% Ficoll-400 MFSW. Prior to imaging, the 4 hpf embryos were fixed, then incubated with peroxidase-labeled anti-DIG antibody, then tyramide signal amplification with rhodamine-tyramide was carried out (see Methods). The video shows a sequence of images taken from the surface of the embryo until to a mid-region. This movie was generated at a frame rate of 2 fps in ImageJ from a 25μm confocal z-stack. Nuclei (blue), rhodamine-tyramide (red).

Supplementary Table S1. List of the shRNA oligonucleotide sequences used for *in vitro* transcription of shRNAs and of the RT-qPCR primer sequences used in this study.

Supplementary Table S2. Embryo survival rate for different shRNA electroporation experiments targeting different genes.

Supplementary Info S1. Protocol: shRNA electroporation in *H. symbiolongicarpus* embryos

**Equipment and Reagents**

Materials and reagents for embryo preparation:

- Artificial seawater (30ppt; Reef Crystals; Catalog Num: 208274)
- Millipore-filtered artificial seawater (MFSW, 30ppt) -- Artificial seawater filtered with a Vacuum Filtration System (PES Membrane, 0.22um, 1000mL, Sterile; Argos Technologies; Catalog Num: BPE2210)--
- Large plastic bins (3 liters, Tri State Plastics; Catalog Num: 195-C)
- Cell strainer -size 70 μm- (white, sterile, individually wrapped; Corning Falcon; Catalog Num: 08-771-2)
- Glass bowls/Culture dishes (Carolina, 4 1/2 in, 250 mL; Catalog Num: 741004)
- Analog Orbital Shaker (Medium, 20 mm Amplitude, 115/230 VAC; Elmi Sky Line; Catalog Num: SHKR-10)
- Stereo Microscope (Stemi 508; Zeiss; Catalog Num: 495009-0018-000)
- Disposable Borosilicate Glass Pasteur Pipets -9 inches- (Fisherbrand; Catalog Num: 13-678-20C)
- Micro Slides (Plain, Culture, Two Depression; Erie Scientific; Catalog Num: 48336-001)
- Rain‑X Original Glass Water Repellent (optional)

Materials and reagents for electroporation mixture preparation:

- Ficoll-400 (PM 400, Type 400; Sigma-Aldrich; Catalog Num: 26873-85-8; F4375)
- Millipore-filtered artificial seawater (MFSW, 30ppt)
- Conical Centrifuge Tubes (15ml; Falcon; Catalog Num: 14-959-49B)
- LP Vortex Mixer (Continuous/Touch Mode, 0 to 3000 rpm; Thermo Fisher Scientific; Catalog Num: 88880017)
- Microcentrifuge tubes (1.5ml; Premium; Fisherbrand; Catalog Num: 05-408-129)
- Nuclease-Free water (Ambion; Catalog Num: AM9937)
- Purified shRNAs

Electroporation equipment:

- ECM 830 Square wave electroporation system with safety stand (Adjustable gap; BTX; Catalog Num: 45-0661) (BTX)
- Electroporation Cuvettes (2mm, Blue Cap, Square Lid, Individually Wrapped, Sterile; Lee Plastic Company; Catalog Num: 4020-02)
- Glass petri dishes (with lid, soda-lime glass, 100 mm × 15 mm; BRAND; Catalog Num: BR455742-10EA)

**Tasks to do prior to an electroporation experiment**

- Make sure the temperature of the experimental lab is ~18ºC. If the temperature is higher the embryos will develop more quickly.
- Prepare all necessary equipment and reagents for the experiment in advance.
- Prepare 15% Ficoll-400 in MFSW (“embryo suspension medium”):
  - In a 15ml conical/falcon tube, dissolve 0.75g Ficoll-400 PM400 in 3.5ml MFSW. Vortex for 2 minutes and shake in a nutator for at least 30 minutes. Adjust the volume to 5ml by adding MFSW. Vortex for 1 more minute and let the tube settle at ~18ºC.

Larger volumes can be prepared, but we recommend 5ml final volume and to prepare fresh 15% Ficoll-400 in MFSW every 2-3 weeks to avoid precipitation and contamination.

15% Ficoll-400 in MFSW is a slightly viscous solution and will initially look cloudy, but it will gradually become transparent within ten minutes.

- Perform the calculations for the electroporation mixtures (including the shRNA dilutions) in advance. Here is an example of a calculation:


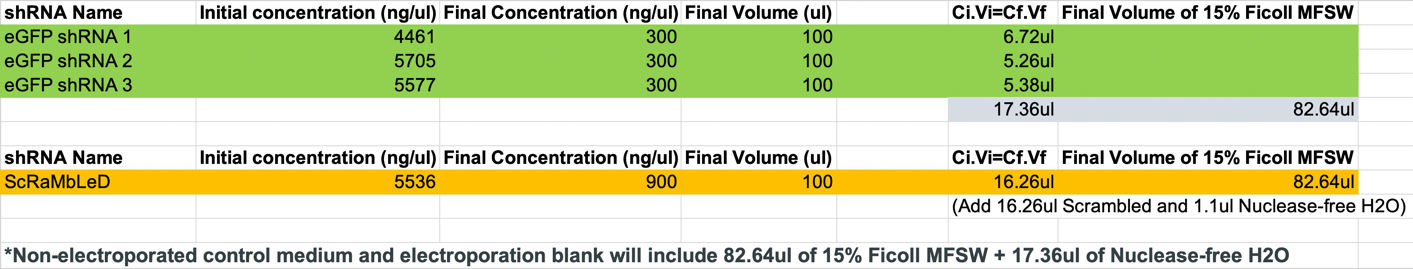


Note: A final volume of **100ul** of electroporation mixture has proven to be optimal for *H. symbiolongicarpus* embryos successful electroporation in a 2mm cuvette.

Note: Always equilibrate salinity (e.g. Using the calculation example above, if you need ~17.4ul of *eGFP* shRNAs and only ~16.3ul of scrambled shRNA to reach the same final concentration, then add ~1.1ul more of Nuclease-free H2O in the scrambled tube to equilibrate final salinities).

- Turn on the ECM 830 system and set up the electroporation conditions to be used.

Optimized parameters for shRNA electroporation in *H. symbiolongicarpus* embryos:

- Voltage (V) = 25V
- Number of pulses = 1
- Pulse length (ms) = 25ms
- Time span between pulses = NA
- Cuvette gap width (1, 2, 4mm) = 2mm

- Prepare the electroporation mixtures.
  - We recommend preparing all electroporation mixtures for a given experiment in 1.5ml sterile microcentrifuge tubes prior to the animals spawning. However, it is better to wait until spawning occurs to add the shRNAs into the electroporation mixtures to avoid wasting shRNAs in case the spawning is not good enough to carry out the experiment. This will also minimize the potential degradation of shRNAs in 15% Ficoll-400 MFSW at 18ºC, since they will be added right before embryo electroporation.

Note: Collect the shRNAs from the -80C freezer and keep them on ice throughout the experiment. shRNAs are generally stable after 2-3 freeze-thaw cycles.

Note: Each experiment should include two types of negative controls, a non-electroporated control and a scrambled shRNA electroporation control. These controls will inform about the fertilization rate of that particular batch, and about the embryo survival upon electroporation with shRNAs that do not target any sequence in the animal’s genome, respectively.

- Optional: Use Rain-X spray to coat the depression slides beforehand, swipe out the excess and let it dry for a few minutes. This helps to make a seawater drop that is easier to manipulate.

**Electroporation protocol**

1. Place *H. symbiolongicarpus* female and male racks inside two separate large plastic bins containing artificial seawater (30ppt) while in the dark, then induce spawning by giving a direct light stimulus.
2. About 1h50min after the light stimulus, both females and males should have spawned eggs and sperm, respectively. Place the racks back into their culture tanks and keep the bins containing the male and female gametes in seawater.
3. Add seawater with sperm into the bin containing seawater with eggs.

Note: Amount of seawater with sperm added depends on the quantity of eggs spawned and the cloudiness of the sperm-containing seawater, which is also dependent on the spawning success.

1. Allow 15 minutes for fertilization to occur.

Note: During this time, add the shRNAs into the appropriate electroporation mixtures and mix by pipetting up and down until solution is homogeneous. Avoid creating bubbles.

Note: The ECM830 system needs a ‘Blank’ step before running a program. Therefore, an extra cuvette with the same amount and proportions of 15% Ficoll-400 MFSW / Nuclease-free water is needed in parallel with your sample for each different electroporation parameters chosen. If all of your samples are using the same electroporation parameters, you will only need to run the Blank step once. We recommend to prepare and run the ‘Blank’ for the chosen electroporation condition during these 15 minutes.

1. Collect the fertilized eggs (one-cell stage embryos) by pouring the seawater through a sterile cell strainer (70um mesh) to catch the eggs. Recover fertilized eggs in a glass bowl filled to 1/3^rd^ with seawater and leave it on an orbital shaker (~90rpm) for ~1 minute. Altogether, this process takes ~5 minutes.
2. Using a Stereo Microscope, collect the fertilized eggs (up to 800 per experimental condition) with a glass Pasteur pipette and place them in different wells of glass depression slides.
3. Focusing on one condition at a time, get rid of most leftover seawater in the well to minimize the dilution of your electroporation mixtures, but never let the embryos dry out. Immediately after removing seawater, add the respective electroporation mixture into the well containing the fertilized eggs and delicately flush the 100ul drop with a glass Pasteur pipette to mix and homogenize.

Note: The fertilized eggs should float in the electroporation mixture thanks to the high concentration of Ficoll-400. If they do not, prepare fresh 15% Ficoll-400 MFSW. The solution should look transparent and clean.

1. Carefully transfer the 100ul drop of electroporation mixture containing the fertilized eggs into an electroporation cuvette (2mm gap) with a glass Pasteur pipette.

Note: Use a black background to see the embryos being transferred into the cuvette.

Note: Avoid creating bubbles.

Note: Avoid touching the walls of the cuvette with the pipette. If fertilized eggs get stuck on the sides of the cuvette chamber they will not be properly electroporated.

1. Delicately flick the cuvette a few times to evenly distribute the fertilized eggs floating inside. Right after this, place the cuvette inside the safety stand connected to the ECM 830 electroporator.
2. Perform the electroporation with the previously chosen parameters by clicking ‘GO’ on the screen.
3. After electroporation is complete, remove the cuvette from the safety stand. Small bubbles should be seen on the walls of the cuvette chamber.
4. Very gently, transfer the electroporated embryos with a glass Pasteur pipette from the cuvette to a large glass petri dish filled with MFSW.

Note: It is crucial to do this step carefully and to evenly distribute the electroporated embryos in the big glass petri dish for them to develop properly. It is useful to pipette a small amount of MFSW from petri dish into the cuvette to gently flush the embryos, collect a small number of embryos, and then gently place in petri dish. Repeat 2-4 times to gently collect all embryos.

Note: For this step it may be useful to have a second person perform to be able to carry out electroporations for several conditions prior to the embryos cleaving.

1. Repeat steps 7-12 for each sample and chosen experimental condition.

Note: *H. symbiolongicarpus* embryos take ~50 minutes to start cleaving when kept at 18ºC, normally allowing time to perform electroporations on up to 6 different samples per experiment.

Note: The electroporation cuvettes can be washed with ultrapure H_2_O and EtOH 100%, let dry for at least 48 hours and be reused again for up to 10 times.

1. Allow the embryos to recover and develop in the large glass petri dishes for several hours without disturbing them.
2. By the end of the day, transfer all healthy, developing embryos to a new glass petri dish. This will ensure an overall better development of the surviving embryos.

Note: Repeat the same procedure of transferring healthy, developing embryos 24 hours after electroporation to ensure proper development of the surviving embryos.

1. Perform detailed phenotypic studies at different timepoints depending on the biological question to be answered.

Supplementary Info S2. shRNA Design

Short hairpin RNAs (shRNAs) are small, synthetic dsRNA molecules connected by a hairpin loop that can be used instead of longer dsRNAs to knock down target genes via RNAi^1^. shRNAs are processed similarly to precursor microRNAs (pre-miRNAs) through the endogenous RNAi pathway of transfected cells. The enzyme Dicer cleaves the loop and converts shRNAs into siRNAs (small interfering RNAs). The resulting siRNAs are unwound into passenger (forward) strands, which are degraded, and guide (reverse) strands, which are incorporated into the RNA-induced silencing complex (RISC). The guide strand-RISC complex binds to the targeted mRNAs, and cleaves them or inhibits their translation^1,2^.

The first step is to find siRNAs targeting your sequence of interest (e.g. *eGFP*). For this step, we recommend the siRNA Wizard web interface from *Invivogen*: <http://www.invivogen.com/sirnawizard/design.php>

- Set motif size to 19 nucleotides.
- Paste the cDNA sequence of your gene of interest (e.g. *eGFP*) and click search.
- The browser will give you all the potential forward strand sequences targeting your gene of interest, the coordinates of where they are located inside your cDNA sequence, and the %GC content of each of them.

**eGFP**

Top of Form

| Sequence | Start | GC% | Design | Scrambled |
| --- | --- | --- | --- | --- |
|  |  |  |  |  |
| GAATTAGATGGTGATGTTA | 49 | 31.58 |  |  |
| **GTGAAGGTGATGCAACATA** | 98 | 42.11 |  |  |
| **GCCAACACTTGTCACTACT** | 171 | 47.37 |  |  |
| GGTTATGTACAGGAAAGAA | 271 | 36.84 |  |  |
| GGTGATACCCTTGTTAATA | 346 | 36.84 |  |  |
| GGAATACAACTATAACTCA | 423 | 31.58 |  |  |
| **GCGTTCAACTAGCAGACCA** | 524 | 52.63 |  |  |
| GCAGACCATTATCAACAAA | 535 | 36.84 |  |  |
| GGCATGGATGAACTATACA | 694 | 42.11 |  |  |
| GCATGGATGAACTATACAA | 695 | 36.84 |  |  |

Bottom of Form

Select three forward strand sequences targeting your gene of interest (our selections are shown here in bold) based on the following criteria:

- Overall GC content between 30-55% (based on the siRNA Wizard web interface from *Invivogen,* sequences with lower GC content seem to be more active than those with higher GC content)
- Low GC content on its 3’ end (helps RISC complex to load the reverse strand instead of the forward strand)
- Avoid sequences with regions of low complexity
- Target genes in the FIRST half of their sequence if possible (to avoid any potential translation of biologically active protein domains even if the mRNA is cleaved by the RISC complex)
  - NOTE: for qPCR primers, target the SECOND half of the sequence, or at least downstream from where the shRNAs are designed.

Next, BLAST the selected forward strands against your species’ genome and transcriptome, if available. A genome assembly and a transcriptome for *H. symbiolongicarpus* male wildtype strain 291-10 is available at: <https://research.nhgri.nih.gov/hydractinia/>

**To minimize potential off-targets, filter out all sequences that have 16 complementary nucleotides or more with non-target genes**.

If available, also BLAST the selected forward strands against your species’ miRNA database. If any of your chosen forward strands yield a match to a miRNA, discard it and select a new one. The miRNA database for *H. symbiolongicarpus* male wildtype strain 291-10 is available at: <https://research.nhgri.nih.gov/hydractinia/>

Once the 3 best forward strands have been selected, use software such as Geneious to create the template of your shRNAs:

1. Generate the reverse strand (the reverse complement of your forward strand)
2. Add the hairpin loop linker in between the forward and reverse strands (in our study, we have used the same linker that was employed in the shRNA design to target genes of the cnidarian *Nematostella vectensis*^3,4^)
3. Include two additional thymidines (TT) at the 3’ end of the template to mimic the endogenous pre-miRNA structure. The total length of the template should be now **49 nucleotides**.

- Here is an example of one shRNA template targeting the *eGFP* gene:

**5’ – GTGAAGGTGATGCAACATATTCAAGAGATATGTTGCATCACCTTCACTT – 3’**

Then, to predict whether your shRNA is going to properly fold *in vitro*, we recommend predicting its secondary structure by using the RNA mfold web server: <http://unafold.rna.albany.edu/?q=mfold>. Click on the ‘RNA folding form’ and paste the complete sequence. Perform prediction keeping all settings as default, click ‘Fold RNA’. Click on ‘Structure 1’ to see the fold.

The RNA mfold web server should predict the formation of a highly stable hairpin structure. If not, discard the sequence and select a new one.

Finally, include the T7 RNA polymerase promoter at the 5’ of your shRNA template. The total length of the template should be now **66 nucleotides**.

- Here is an example of a DNA template for *in vitro* transcription of an shRNA targeting the *eGFP* gene:

**5’ – TAATACGACTCACTATAGTGAAGGTGATGCAACATATTCAAGAGATATGTTGCATCACCTTCACTT – 3’**

A dsDNA template is needed for the *in vitro* transcription of shRNAs. Thus, a reverse complement of the 66 nucleotide-long sequences also needs to be designed. Both forward and reverse ssDNA oligonucleotides can then be ordered.

**Examples**

**Construct *eGFP* shRNA #1**      siRNA GC%: 42.11      Position: 98

Hairpin structure

5' GTGAAGGTGATGCAACATATTCAAGAGATATGTTGCATCACCTTCACTT 3'

- No hits found either in the Transcriptome or in the Genome of *H. symbiolongicarpus* that have complementarity of 16 nucleotides or more to the forward strand
- No hits found in the *H. symbiolongicarpus* miRNA database


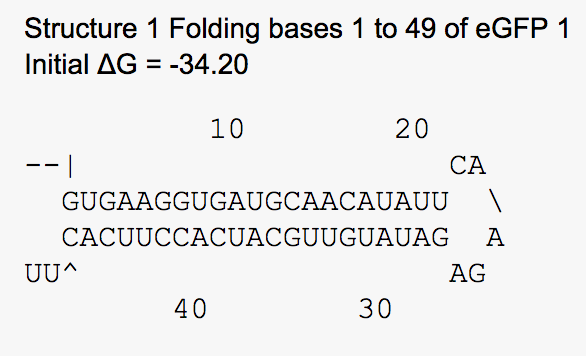


Oligo eGFP #1 (For)

5' TAATACGACTCACTATAGTGAAGGTGATGCAACATATTCAAGAGATATGTTGCATCACCTTCACTT 3'

Oligo eGFP #1 (Rev)

5' AAGTGAAGGTGATGCAACATATCTCTTGAATATGTTGCATCACCTTCACTATAGTGAGTCGTATTA 3'

**Construct *eGFP* shRNA #2**      siRNA GC%: 47.37      Position: 171

Hairpin structure

5' GCCAACACTTGTCACTACTTTCAAGAGAAGTAGTGACAAGTGTTGGCTT 3'

- No hits found either in the Transcriptome or in the Genome of *H. symbiolongicarpus* that have complementarity of 16 nucleotides or more to the forward strand
- No hits found in the *H. symbiolongicarpus* miRNA database


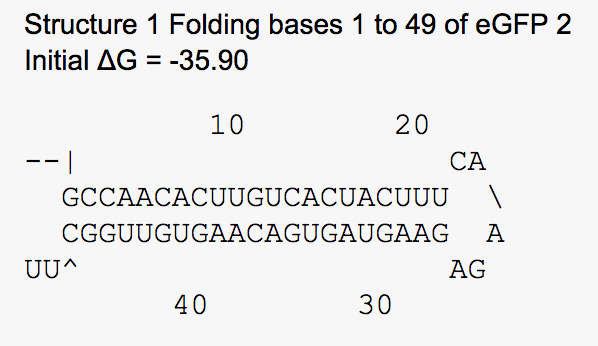


Oligo eGFP #2 (For)

5' TAATACGACTCACTATAGCCAACACTTGTCACTACTTTCAAGAGAAGTAGTGACAAGTGTTGGCTT 3'

Oligo eGFP #2 (Rev)

5' AAGCCAACACTTGTCACTACTTCTCTTGAAAGTAGTGACAAGTGTTGGCTATAGTGAGTCGTATTA 3'

**Construct *eGFP* shRNA #3**      siRNA GC%: 52.63      Position: 524

Hairpin structure

5' GCGTTCAACTAGCAGACCATTCAAGAGATGGTCTGCTAGTTGAACGCTT 3'

- No hits found either in the Transcriptome or in the Genome of *H. symbiolongicarpus* that have complementarity of 16 nucleotides or more to the forward strand
- No hits found in the *H. symbiolongicarpus* miRNA database


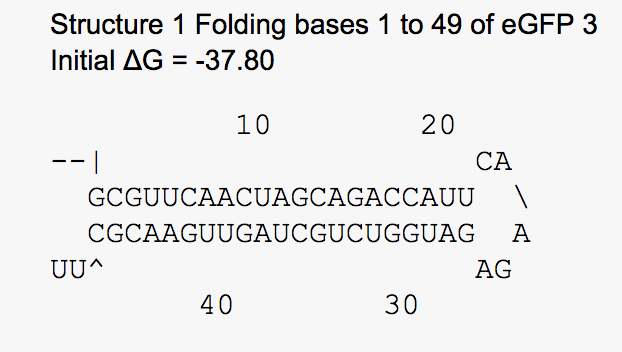


Oligo eGFP #3 (For)

5' TAATACGACTCACTATAGCGTTCAACTAGCAGACCATTCAAGAGATGGTCTGCTAGTTGAACGCTT 3'

Oligo eGFP #3 (Rev)

5' AAGCGTTCAACTAGCAGACCATCTCTTGAATGGTCTGCTAGTTGAACGCTATAGTGAGTCGTATTA 3'

**References**

1. Rao, D. D., Vorhies, J. S., Senzer, N. & Nemunaitis, J. siRNA vs. shRNA: Similarities and differences. *Adv. Drug Deliv. Rev.* **61**, 746–759 (2009).

2. Cullen, B. R. RNAi the natural way. *Nat. Genet.* **37**, 1163–1165 (2005).

3. Karabulut, A., He, S., Chen, C.-Y., Mckinney, S. A. & Gibson, M. C. Electroporation of short hairpin RNAs for rapid and efficient gene knockdown in the starlet sea anemone, *Nematostella vectensis*. *Developmental Biology*. **448**, 7-15 (2019).

4. He, S. *et al.* An axial Hox code controls tissue segmentation and body patterning in *Nematostella vectensis*. *Science.* **1380**, 1377–1380 (2018).
